# Activation and functional connectivity of cerebellum during reading and during arithmetic in children with combined reading and math disabilities

**DOI:** 10.3389/fnins.2024.1135166

**Published:** 2024-04-29

**Authors:** Sikoya M. Ashburn, Anna A. Matejko, Guinevere F. Eden

**Affiliations:** Center for the Study of Learning, Department of Pediatrics, Georgetown University Medical Center, Washington, DC, United States

**Keywords:** activation, connectivity, reading, arithmetic, cerebellum, dyslexia, dyscalculia, children

## Abstract

**Background:**

Reading and math constitute important academic skills, and as such, reading disability (RD or developmental dyslexia) and math disability (MD or developmental dyscalculia) can have negative consequences for children’s educational progress. Although RD and MD are different learning disabilities, they frequently co-occur. Separate theories have implicated the cerebellum and its cortical connections in RD and in MD, suggesting that children with combined reading and math disability (RD + MD) may have altered cerebellar function and disrupted functional connectivity between the cerebellum and cortex during reading and during arithmetic processing.

**Methods:**

Here we compared Control and RD + MD groups during a reading task as well as during an arithmetic task on (i) activation of the cerebellum, (ii) background functional connectivity, and (iii) task-dependent functional connectivity between the cerebellum and the cortex.

**Results:**

The two groups (Control, RD + MD) did not differ for either task (reading, arithmetic) on any of the three measures (activation, background functional connectivity, task-dependent functional connectivity).

**Conclusion:**

These results do not support theories that children’s deficits in reading and math originate in the cerebellum.

## 1 Introduction

Reading and math skills are acquired in parallel during childhood through formal instruction (Purpura et al., 2019). However, reading disability (RD or developmental dyslexia) and math disability (MD or developmental dyscalculia) can manifest despite normal intellectual ability and appropriate instruction, leading to deleterious academic and personal outcomes. RD is a difficulty in acquiring accurate and fluent reading (Lyon et al., 2003; Vellutino et al., 2004) and impacts 5–12% of children (Katusic et al., 2001). The cause of RD is thought to be poor phonological awareness, which is the ability to isolate and manipulate sounds in words (Stanovich, 2016), and underdeveloped orthographic processing (Badian, 1995). These factors are thought to impede the ability to map phonemes onto graphemes, and to recognize visual word forms, respectively (Lyon et al., 2003; Vellutino et al., 2004). In contrast, MD, is characterized by poor computational skills and arithmetic fact retrieval (Castaldi et al., 2020) and impacts 3–6% of the population (Gross-Tsur et al., 2008). MD is thought to be caused by poor numerical magnitude processing, which is the ability to represent and manipulate numerical quantities (Butterworth, 2010; Piazza, 2010). This leads to difficulties learning and retrieving arithmetic facts from long-term memory (Geary, 2004; Peters and De Smedt, 2018). Mainstream theories describe aberrant function of left-hemisphere perisylvian regions during phonological and orthographic processing in RD (Maisog et al., 2008; Gabrieli, 2009; Richlan et al., 2011; Linkersdörfer et al., 2012; Eden et al., 2015), and aberrant function of bilateral fronto-parietal regions during magnitude and numerical processing in MD (Ashkenazi et al., 2013; Peters and De Smedt, 2018; Martinez-Lincoln et al., 2023; Tablante et al., 2023). However, separate lines of research also implicate the cerebellum as a cause of RD (Nicolson et al., 2001; Nicolson and Fawcett, 2019) and MD (Vandervert, 2017), but support of these models is mixed. In the current study we focus on children with combined RD and MD, reasoning that if the cerebellum is required for successful reading and arithmetic, and aberrations of the cerebellum lead to RD or MD, those with combined RD and MD are most likely to have altered cerebellar function during reading and during arithmetic. Indeed, RD and MD have a high rate of co-occurrence, with 28–64% of children with RD also having MD (Willcutt et al., 2013). Further, while prior studies into these learning disabilities have mostly employed a whole-brain analysis approach to capture differences in activity in RD and in MD, here we focus the analyses specifically on the cerebellum and its cortical connections.

The Cerebellar Deficit Hypothesis of Dyslexia posits that the cerebellum is important for fluent reading through its connections with frontal cortical regions involved in articulatory and phonological processing; and that impaired cerebellar function during development in RD leads to dysfunctional connections between the cerebellum and these frontal regions (Nicolson et al., 2001). This theory aims to account for the widely described weakness in phonological processing in RD, as well as for deficits more directly attributed to the cerebellum, such as poor skill automatization and timing. This theory has undergone revision, most recently referred to as the Delayed Neural Commitment Hypothesis (Nicolson and Fawcett, 2019). A similar yet separate hypothesis posits that the cerebellum and connecting frontal and parietal systems are involved in mathematics via sequence (pattern) detection, and automatization of number manipulation, as well as verbal working memory, executive control, inner speech and visual–spatial learning (Vandervert, 2017); and that dysfunctional connections between the cerebellum and frontal and parietal systems lead to MD. Together these two theories deem the cerebellum and its connections with the cortex to be critical for successful reading and, separately, for successful math, thereby independently implicating the cerebellum in RD and MD. While the theories about cerebellar involvement in reading (Nicolson et al., 2001) and arithmetic (Vandervert, 2017) were developed separately, they describe several functions attributed to the cerebellum that could be important for both skills in cognitive (working memory), linguistic (phonological processing) and motor (articulation) domains, as well as the more ubiquitous phenomenon of automatization. If the two theories describing cerebellar impairment and compromised cerebellar-cortical connections in these learning disabilities are correct, one would hypothesize activity in the cerebellum, and functional connectivity between the cerebellum and specific cortical regions, to be altered during both reading and arithmetic in children with RD + MD. The location for such differences within the cerebellum would be indicative of the mechanism by which cerebellar dysfunction leads to RD or MD, and whether or not they are the same for RD and MD.

Some studies have reported differences in the cerebellum in children with RD. A series of studies involving reading of Chinese characters in typically-reading children have shown activation of right lobule VI of the cerebellum (Li et al., 2022); and functional connectivity between right lobule VI of the cerebellum and left supramarginal gyrus that was related to participants’ rapid automatized naming skills (Ang et al., 2020). Further, lobule VI of the cerebellum was found to be more active in those with RD compared to controls (Feng et al., 2017). However, another study did not find differences in activation of the cerebellum between good and poor readers, but did report between-group differences in functional connectivity between right lobule VI and left angular gyrus (Li et al., 2020). Importantly, if there are differences in the cerebellum related to reading or math disability, they are best investigated in participants with both learning disabilities performing both reading and math tasks in the same study; and to investigate activity and functional connectivity simultaneously. Only in this way will it become clear if differences due to poor reading or math skills converge on the same region(s) of the cerebellum, thereby shedding light on the potential mechanisms by which the cerebellum affects these important academic skills.

In typically developing children and adolescents (henceforth we use children, noting that studies of children often also include adolescents), reading in alphabetic languages is associated with activation of a left hemisphere network involving left frontal, posterior parietal, and occipital-temporal regions as demonstrated by a meta-analysis (Martin et al., 2015). The cerebellum, however, is not traditionally considered to be integral to children’s reading. A few studies included in this meta-analysis do report activation in the cerebellum during reading (Booth et al., 2001; Gaillard et al., 2003; Hoeft et al., 2006; Noble et al., 2006; Rimrodt et al., 2009). When it comes to children with dyslexia, relatively less activity during reading tasks in alphabetic languages has been revealed by meta-analysis in bilateral inferior parietal and left occipital cortices, and relatively more activity in left frontal cortex (Richlan et al., 2011). Very few studies in this meta-analysis implicate the cerebellum in dyslexia, with two studies finding relatively more cerebellar activation in RD (Temple et al., 2001; Meyler et al., 2008). Arithmetic problem solving in children has been shown via meta-analysis to rely on a set of bilateral fronto-parietal brain regions (Arsalidou et al., 2018). Again, while the cerebellum was not identified as a common contributor, some of the studies included in Arsalidou et al., did report the cerebellum to be active during arithmetic in typical children (Meintjes et al., 2010; de Smedt et al., 2011; Mondt et al., 2011; Ashkenazi et al., 2012; Du et al., 2013; Qin et al., 2014; Peters et al., 2016). There have been few comparisons between children with and without MD and no meta-analyses, but recently two meta-analyses combining children and adults have been published and both revealed differences in right parietal lobe in MD and did not implicate the cerebellum in MD (Martinez-Lincoln et al., 2023; Tablante et al., 2023). Only two of the original studies contributing to both of these meta-analyses reported altered cerebellar activation during an arithmetic task. One found relatively less activation in the cerebellum in children with MD (Ashkenazi et al., 2012), while the second one reported more (Iuculano et al., 2015). Interestingly, anatomical differences in the cerebellum have been reported in children with RD (Stoodley, 2014) as well as children with MD (Rykhlevskaia, 2009) relative to controls.

In sum, despite theories implicating the cerebellum in RD and in MD, the cerebellum is rarely found to be active during reading or arithmetic in typically-developing children, or to differ in children with learning disabilities in reading or math. Only a few functional neuroimaging studies have explicitly examined the role of the cerebellum during reading in children with RD, and none during arithmetic in MD. Here we test for functional differences in the cerebellum during reading and during arithmetic in children with RD + MD relative to controls. As the current evidence for cerebellar deficit theories in RD and MD is weak, it is plausible that our results will not support these theories. A better understanding of the role of the cerebellum in RD and MD is important for devising brain-based models of learning disabilities and has implications for treatment. Here we used fMRI to compare typically-developing children to children with RD + MD during reading (Study 1) and during arithmetic (Study 2). For Study 1, we examined the cerebellum for (i) brain activity during single word processing, (ii) background functional connectivity (Norman-Haignere et al., 2012) between the cerebellum and cortical regions known to be involved in reading, and (iii) reading-related functional connectivity between the cerebellum and cortical regions known to be involved in reading. For Study 2, we used an arithmetic task and followed the same methodological framework as Study 1, this time testing for (i) cerebellar activation during arithmetic processing, as well as (ii) background and (iii) arithmetic-modulated functional connectivity between the cerebellum and cortical regions known to be involved in arithmetic. For all analyses, we report within-group results for the Control group and for the group with RD + MD, and to address the primary research question of a cerebellar deficit in these learning disabilities we tested for between-group differences.

## 2 Materials and methods

### 2.1 Participants

All children were recruited as part of our program of research on learning disabilities, either from the community or from a school that specializes in teaching children with learning disabilities. All were monolingual, native English speakers. Participants were given informed consent prior to beginning the study and all protocols were approved by the Georgetown University Institutional Review Board. Subsets of these participants were included in prior fMRI publications (Olulade et al., 2013, 2015; Evans et al., 2014; Ashburn et al., 2020).

Behavioral assessments included the Wechsler Abbreviated Scale of Intelligence (WASI; Wechsler, 1999) and the Woodcock-Johnson III Tests of Achievement (WJ-III; Woodcock et al., 2001). To be in the study, all participants had to have a standard score for Intelligence Quotient (IQ) on the WASI of 80 or above. The WJ-III battery was used to assess single real-word reading ability (Word Identification), pseudo-word reading ability (Word Attack), simple fact retrieval (Math Fluency), and more complex mathematical functions (Calculation). A standard score of 100 represents the 50^th^ percentile and a score between 85 and 115 (one standard deviation above or below the mean) is considered to be the average range of performance. Children in the Control group were required to have a standard score above 92 on both the real- and pseudo- word reading subtests as well as above 92 on both the math fluency and calculation subtests of the WJ-III (28 out of 33 met these criteria). This ensured that the Control group was well within or above the average range for reading or math. Children with RD + MD were selected from a larger group of children with learning disabilities based on a standard score of 85 (16th percentile) or below on either, or both, the real- or pseudo-word reading subtest, as well as a standard score of 85 or below on either (or both) the math fluency and calculation subtests of the WJ-III (30 out of 92 met these criteria).

Children with anatomical anomalies observed on the structural MRI or those with excessive head movement in the fMRI scans (described below) were excluded. For Study 1, nine children were excluded, leaving 23 children in the Control group (13 females, 10 males, mean age = 9.7 years, standard deviation [SD] = 1.8) and 26 in the group with RD + MD (12 females, 14 males, mean age = 10.3, SD = 1.4). The groups did not differ significantly in age. However, because they differed in Verbal and Performance IQ, Full IQ was used as a covariate of no interest when analyzing the fMRI data for between-group differences on the reading task. As expected, the RD + MD group had significantly lower reading and math scores than the Control Group. All participants except one Control participant were right-handed. Group characteristics are provided in Supplementary Table 1.

For Study 2, the arithmetic task was not acquired for all participants that were in Study 1, and, after excluding one child due to head movement and another due to incomplete brain coverage, Study 2 had 16 children in the Control group (6 females, 10 males, mean age = 10.1 years, SD = 2.0) and 14 in the group with RD + MD (6 females, 8 males, mean age = 10.8, SD = 1.3). As in Study 1, the groups did not differ significantly in age, but they again differed in Verbal and Performance IQ, and therefore Full IQ was used as a covariate of no interest in the between-group analyses of the arithmetic task. As expected, the RD + MD group again had significantly lower reading and math scores than the Control Group. All participants in Study 2 were right-handed. Group characteristics are provided in Supplementary Table 2.

### 2.2 fMRI tasks

**Figure 1:**
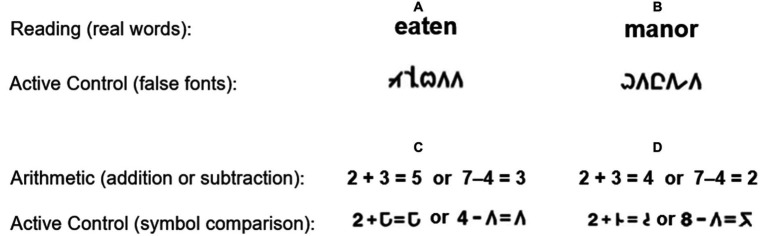
Examples of stimuli in Study 1 **(A,B)** and Study 2 **(C,D)**.

In Study 1, participants performed an implicit reading task (Price et al., 1996; Ashburn et al., 2020), consisting of visually presented real word and false font conditions. Real word stimuli were single five-letter, low frequency words used for the Reading task. False font stimuli were used for the Active Control condition and were created by manipulating the letters from the real word stimuli to create new, unfamiliar characters. False font strings were matched to real words for both length and location of ascenders and descenders. As such the number of elements and angles are similar across the Reading (real words) and Active Control (false fonts) conditions. Participants were instructed to indicate whether the visually presented stimulus had a “tall” character. Participants responded by pressing a button in their right hand if a tall feature was present (e.g., Figure 1A) and pressing a button in their left hand if no such feature was present (e.g., Figure 1B) in the real word or false font stimuli. They were instructed to respond as accurately and quickly as possible. Reading and Active Control stimuli were presented in separate blocks, always alternating with a block of fixation. During Fixation blocks children were instructed to keep their eyes on the cross hair in the center of the screen. We examined Reading > Fixation as a way to gauge general activation to the task and Reading > Active Control to identify activity specific to single word processing.

Each participant completed two runs and each run consisted of two blocks of each task condition (Reading and Active Control), with 10 stimuli per block. The inter- stimulus trial was 4.2 s and each task block had a duration of 42 s while interleaving Fixation blocks had a duration of 18 s blocks. Therefore, the overall length of the run was 4 min and 27 s. The number of brain volumes acquired was the same for the Reading (real words), Active Control (false fonts), and Fixation conditions (28 volumes each per run). Both runs were used for all participants except for three Control participants, where one of the two runs was removed due to excessive motion. At the conclusion of the actual scanning session, a pencil-and-paper test was performed in which participants were asked whether they had seen a given stimulus during the scans (as in Turkeltaub et al., 2003). There were 40 targets and 40 foils, for each condition.

For Study 2, participants performed a single-digit arithmetic verification task (Evans et al., 2014, 2016), which included addition and subtraction blocks. The task was a two-operand equation with a single-digit answer, and participants indicated with a right or left button press whether the math problem was correct (e.g., 2 + 3 = 5 or 7–4 = 3) or incorrect (e.g., 2 + 3 = 4 or 7–4 = 2) as shown in Figure 1. Both addition and subtraction had Active Control conditions where one of the components of the equation on either side was replaced by a symbol (symbol comparison). In this instance, children indicated whether the symbols on either side of the equal sign were the same (e.g., Figure 1C) or different (e.g., Figure 1D). Each condition (addition, addition active control, subtraction, and subtraction active control) consisted of 10 unique stimuli. Each block consisted of 50% correct and 50% incorrect problems that were randomized within each block. We examined Arithmetic > Fixation as a way to gauge general activation to the task and Arithmetic > Active Control to identify activity specific to arithmetic processing.

Each participant completed two runs and each run consisted of two blocks of each task condition, Arithmetic (addition or subtraction) and Active Control (symbol comparison), with 10 stimuli per block. The task blocks, length of the run and number of brain volumes acquired per condition (28 volumes of each, Arithmetic, Active Control and Fixation) were analogous to those used for Study 1. Both runs were used for all participants except for one Control and three RD + MD participants.

Prior to the scanning session all participants practiced the task in a mock scanner to become habituated to all of the tasks and to the scanning environment. We used Presentation software (Neurobehavioral Systems Inc., Albany, CA, United States) for stimulus presentation and recording responses. We collected reaction time (RT) and accuracy for all tasks. RT and accuracy were compared between the groups using a two-sample student *t*-test (Supplementary Tables 3, 4). For Study 1, one Control and one RD + MD participant did not have in-scanner performance data due to a technical malfunction while for Study 2 all participants had in-scanner performance data.

### 2.3 Image acquisition

All scans were acquired at the Center for Functional and Molecular Imaging at Georgetown University on a 3 T Siemens scanner. Structural T1 images were acquired using FOV = 256, phase = 250, slices = 160, and slice resolution = 1 mm, resulting in 1.0 × 1.0 × 1.0 mm voxels. Functional images were obtained with a T2*-weighted echo planar imaging sequence using Flip Angle = 90°, TR = 3 s, TE = 30 ms, and 50 axial slices (2.8 mm with a 0.2 mm gap), FOV = 192 mm, in-plane resolution =64×64, resulting in 3 mm cubic voxels. Three RD + MD children in Study 1 and four RD + MD children in Study 2 were collected after an upgrade and this was included as a covariate of no interest for all between-group comparisons. Functional images had complete coverage of the cortex and cerebellum.

### 2.4 Data analysis

Measures for Study 1 can be considered in three parts: (i) cerebellar activity during word processing in comparison to Fixation (Reading > Fixation) and in comparison to the specific Active Control false font task (Reading > Active Control); (ii) background functional connectivity (Norman-Haignere et al., 2012); and (iii) and task-dependent (generalized psychophysiological interactions, gPPI) functional connectivity. Background functional connectivity (FC) analysis probes how the cerebellum may be intrinsically connected to cortical regions independent of the task. The task related gPPI FC analysis distinguishes whether these functional connections are specific to word processing. For all analyses, we generated within-group and between-group maps. We constrained the analyses to the cerebellum, as described in detail below.

Using a similar approach to Study 2, we examined (i) cerebellar activity during arithmetic processing in comparison to Fixation (Arithmetic > Fixation) and to the specific Active Control task (Arithmetic > Active Control); (ii) task-independent background FC; and (iii) task-dependent gPPI FC during arithmetic task.

#### 2.4.1 Preprocessing

For all analyses, data were individually inspected for gross artifacts and to ensure full cerebellum coverage. The preprocessing steps for both Study 1 and Study 2 were completed with Statistical Parametric Mapping, version 12 (SPM12; Welcome Department of Cognitive Neurology, London). The toolboxes SUIT (Diedrichsen et al., 2009) and Voxel Based Morphometry segmentation (Ashburner and Friston, 2000) were used for activation and functional connectivity analyses, respectively. The first five functional images of each run were discarded. Functional images were slice-time corrected, realigned, and co-registered to the anatomical data.

All data were corrected for head movement using ArtRepair (ART^1^; adjusted in-house). Time points with scan-to-scan motion greater than 0.75 mm (25% of the voxel size) were regressed out during statistical analysis. The percentage of scans regressed out in this way did not differ between the two groups for either Study 1 or Study 2 (*p* > 0.05). A participant’s data were entirely excluded from the analysis if: (i) more than 20% of the scans (averaged across the two runs) exceeded the 0.75 mm motion threshold, (ii) greater than 25% of scans exceeded the 0.75 mm threshold in either run, or percent global signal change was greater than 5%.

#### 2.4.2 Functional activation analyses

After preprocessing, we ran first-level general linear model analysis on the functional data, thereby generating contrast images for each subject (Reading > Fixation, and Reading > Active Control for Study 1; and Arithmetic > Fixation, and Arithmetic > Active Control for Study 2). We then used SUIT to isolate the cerebellum. For this step, we generated a cerebellar mask for each participant, which was quality controlled and manually corrected by overlaying the mask onto the T1-anatomical image within MRICron (Rorden et al., 2007). Careful attention was given to the border between the cerebellum and cerebrum to avoid including voxels in the adjacent inferior occipital or temporal cortex. Next, we normalized the anatomical image into SUIT space and used the resulting deformation field to transform the fMRI data into SUIT space. Lastly, these normalized images were smoothed with a 4x4x4-mm full-width height maximum Gaussian kernel. Both within- and between-group significance was determined by height threshold = 0.001, *p* < 0.05 FWE-corrected.

In addition to analyzing the cerebellum as a whole, for both Study 1 and Study 2 we conducted analyses using sub-regions within the cerebellum implicated in reading (Stoodley et al., 2012; Martin et al., 2015) and arithmetic (King et al., 2019). Specifically for right and left lobule VI, crus I, crus II, and lobule VIIb masks were defined within the SUIT atlas (Diedrichsen et al., 2009) and used Small Volume Correction (SVC) at the second level to conduct the region of interest (ROI) analyses for each of the eight sub-region. Both within- and between-group significance was determined by height threshold = 0.001, and we used a Bonferroni-correction to account for the use of multiple ROIs, such that the adjusted threshold for significance was p-FWE-Bonferroni <0.00625. Within this article, we use the term ‘cerebellar sub-regions’ to refer these ROI in the analysis of activation. These frequentist analyses were then followed by Bayesian analyses to examine the strength of evidence for the null versus alternative hypotheses. Using the same values extracted from the eight cerebellar sub-regions, we used the beta values for each task versus control comparison to establish evidence for the null hypothesis versus the alternative hypothesis when comparing the groups. Specifically, for Study 1 we examined (i) cerebellar activity during word processing in comparison to fixation (Reading > Fixation) and in comparison to false fonts (Reading > Active Control); and for Study 2 we examined (i) cerebellar activity during arithmetic processing in comparison to fixation (Arithmetic > Fixation) and in comparison to symbol comparison (Arithmetic > Active Control). The analyses were conducted using Bayesian Independent Samples *t*-test in the open statistical software program JASP (Version 0.9.2; JASP Team, 2023).

#### 2.4.3 Functional connectivity analyses

For both Study 1 and Study 2, the preprocessed functional data were segmented using Voxel Based Morphometry (Ashburner and Friston, 2000), and normalized to MNI space. We then used CONN toolbox 16.b (Whitfield-Gabrieli and Nieto-Castanon, 2012) to perform background as well as task-dependent connectivity analyses. Within CONN toolbox, we performed denoising with simultaneous regression of temporal confounding factors as well as temporal filtering on unsmoothed functional data. The temporal confounding factors included six head position parameters, a vector to indicate whether a particular scan was preceded by our 0.75 mm threshold (whereby scans preceded by inter-scan head motion <0.75 mm received a 0 and scans preceded by inter-scan head motion greater than or equal to 0.75 mm received a 1), and block conditions convolved with a canonical hemodynamic response function. For Study 1, the modeled block conditions included Reading (real words), Active Control (false fonts), and Fixation, whereas for Study 2, the block conditions included Arithmetic (addition and subtraction), Active Control (symbol comparison), and Fixation. CONN toolbox also estimated principal components from subject-specific white matter and CSF masks, derived from the VBM segmentation step above. Five principal components were created for both white matter and CSF per subject.

First, we performed a *background functional connectivity*. This approach regresses out the effects of task blocks from a run of fMRI data, to generate a measure of intrinsic brain connectivity. Thus, for Study 1 we regressed the effects of Reading, Active Control, and Fixation. Likewise, for Study 2, we regressed the effects of Arithmetic, Activate Control, and Fixation. For both studies, we applied a low band-pass filter (0.008 to 0.09 Hz). First-level analysis was performed using GLM, HRF weighting, and bivariate correlation parameters for ROI-to-ROI analysis. (More details on ROIs below.) For each set of right and left cerebellar ROIs (lobule VI, crus I, crus II, lobule VIIb) we performed first-level analyses, while cortical ROIs remained the same across all analyses. Of note, cerebellar ROIs for FC analyses will be referred to as ‘cerebellar seeds.’ Second-level analysis was performed on each individual cerebellar seed, that is, for example, right lobule VI seed was tested against nine cortical target regions.

Second, we used gPPI regression analyses to provide insight into *task-dependent* cerebellar connectivity, i.e., connectivity modulated by either word processing (Study 1) or arithmetic processing (Study 2). For these analyses, we applied a high band-pass filter (0.008 Hz to Inf Hz). First-level analysis was performed using gPPI and bivariate regression parameters for ROI-to-ROI analysis. This analysis accounts for each task condition in a regression model, i.e., Reading and Active Control in Study 1 and Arithmetic and Active Control in Study 2. Second-level analyses were performed for each individual cerebellar seed for the contrast of Reading > Active Control for Study 1 and the contrast of Arithmetic > Active Control for Study 2.

**Figure 2:**
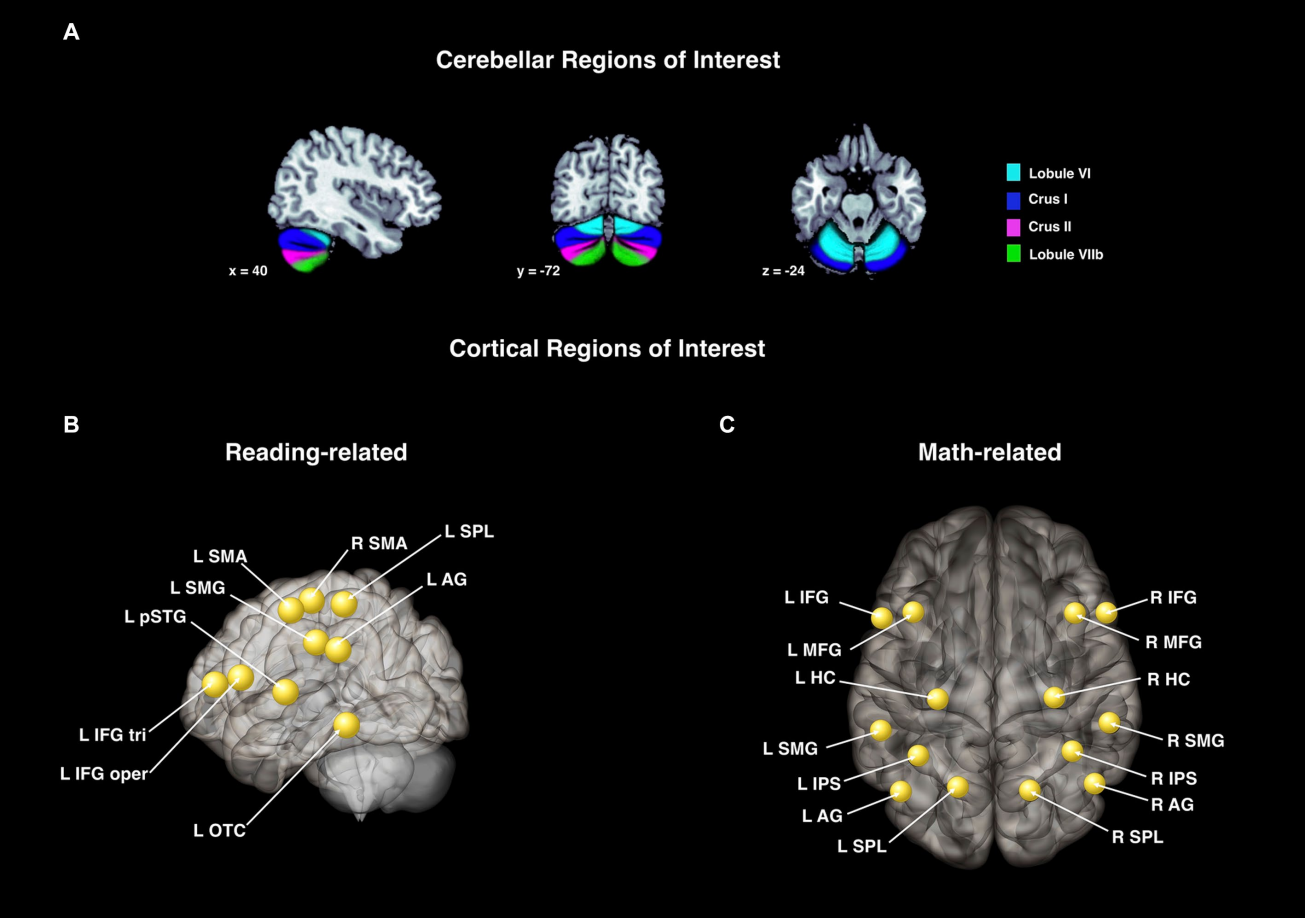
Cerebellar and cortical regions of interest used for the activation and connectivity analyses. These ROIs were used for the activation and connectivity analyses for Study 1 **(A,B)** and Study 2 **(A,C)**. **(A)** Cerebellar regions chosen based on the literature and defined with the SUIT atlas (Diedrichsen et al., 2009). These were used in the activation analysis (cerebellar sub-regions) and again in the connectivity analyses (cerebellar seed regions). Cortical target regions for the functional connectivity analyses in Study 1 **(B)** and Study 2 **(C)**. Regions are shown as spheres but all represent anatomical regions.

Cerebellar *seed regions* for the connectivity analyses in both background and task-dependent analyses were the same eight cerebellar sub-regions as those described above for the activation analyses, chosen based on the literature: left and right lobule VI, crus I, crus II, and lobule VIIb (Figure 2A).

Cortical *target regions* for Study 1 were chosen based on the traditional reading network as defined by Pugh et al. (2001) and the meta-analysis by Martin et al. (2015). Specifically, we selected the following nine regions within CONN (Harvard-Oxford atlas; Desikan et al., 2006): left inferior frontal gyrus *pars triangularis* (IFG tri), inferior frontal gyrus *pars opercularis* (IFG oper), posterior superior temporal gyrus (pSTG), superior parietal lobule (SPL), supramarginal gyrus (SMG), angular gyrus (AG), occipital-temporal cortex (OTC), and left and right supplementary motor area (SMA). Of note, these regions were anatomically defined, but are represented by CONN as spheres in the resultant figures. Visualization of these ROIs can be found in Figure 2B, and are the same as those reported in Ashburn et al. (2020) with the addition of the SMA.

Cortical *target regions* for Study 2 were chosen based on a review of neuroimaging studies on arithmetic (Peters and De Smedt, 2018). These 14 cortical target regions for the arithmetic network (Figure 2C) included left and right: hippocampus (HC), superior parietal lobules (SPL), intraparietal sulcus (IPS), angular gyrus (AG), supramarginal gyrus (SMG), inferior frontal gyrus (IFG), and middle frontal gyrus (MFG). Except for the middle frontal gyri, these were all created using the cytoarchitectonic maps provided in the Anatomy Toolbox (Eickhoff et al., 2005, 2007). However, a clear delineation of the middle frontal gyri was not included in the Anatomy Toolbox. Thus, the middle frontal gyri were created from the probabilistic Harvard-Oxford Atlas in FSL. Any overlap with the inferior frontal gyri ROI was removed.

Both within- and between-group significance for background functional connectivity and task-dependent functional connectivity was determined with p-FDR =0.05, seed-level correction, two-sided statistic. CONN toolbox was also used to visualize results. Spheres were overlaid onto these images to optimize the visibility of the seed and target regions.

## 3 Results

### 3.1 Study 1: word processing

#### 3.1.1 Behavioral measures

Accuracy and response time for the Control and RD + MD groups for word processing are shown in Supplementary Table 3. Most relevant to our fMRI activation analyses is that there were no significant differences between the Control group and RD + MD group for accuracy or response time when comparing the difference between the Reading and Active Control conditions for these performance measures.

The pencil-and-paper test used to assess the participants’ familiarity with the stimuli after completion of the scan found that both groups performed significantly above chance (*p* < 0.05) when identifying real word but not false font stimuli, indicating that participants had processed the word stimuli during the scan.

#### 3.1.2 Word processing: activation analysis constrained to (i) the whole cerebellum and (ii) cerebellar sub-regions (left and right lobule VI, crus I, crus II, lobule VIIb)

The reporting of significant results for both within- and between-groups are based on a height threshold = 0.001, p < 0.05 FWE-corrected (for whole cerebellum) and *p* < 0.00625 FWE-Bonferroni-corrected (for cerebellar sub-regions).

##### 3.1.2.1 Control group

**Figure 3:**
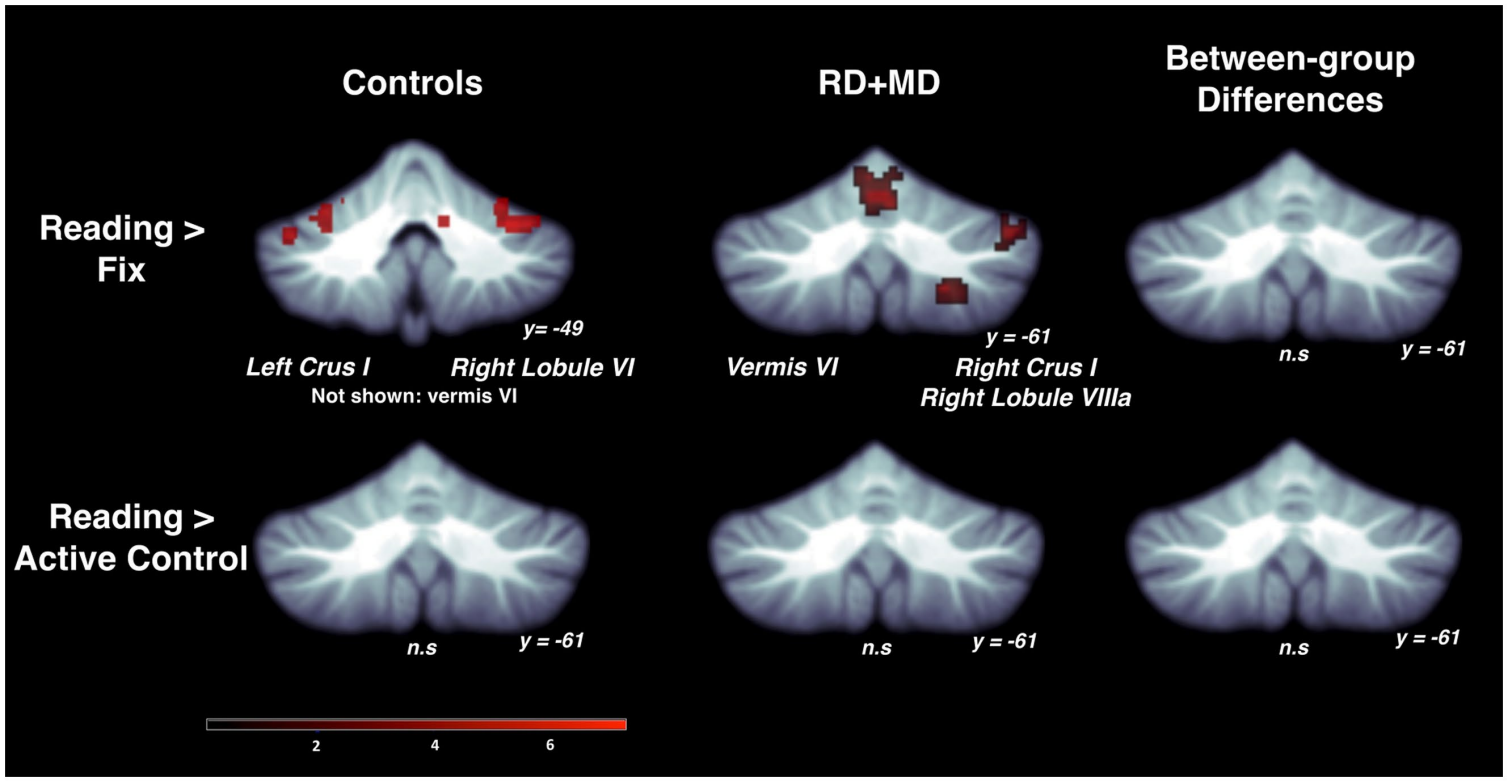
Activation during reading in Control and RD + MD groups using whole cerebellum analyses. Reading > Fixation contrast, and Reading > Active Control contrast. Significant activation in vermis VI (not shown), left crus I, and right lobule VI in Control group, height threshold *p* < 0.001, *p* < 0.05 FWE-corrected. Also, activation in vermis VI extending into vermis VI, right crus I, and right lobule VIIIa in the RD + MD group. No significant activation for Reading > Active Control for either group, and no between-group differences for either contrast.

**Figure 4:**
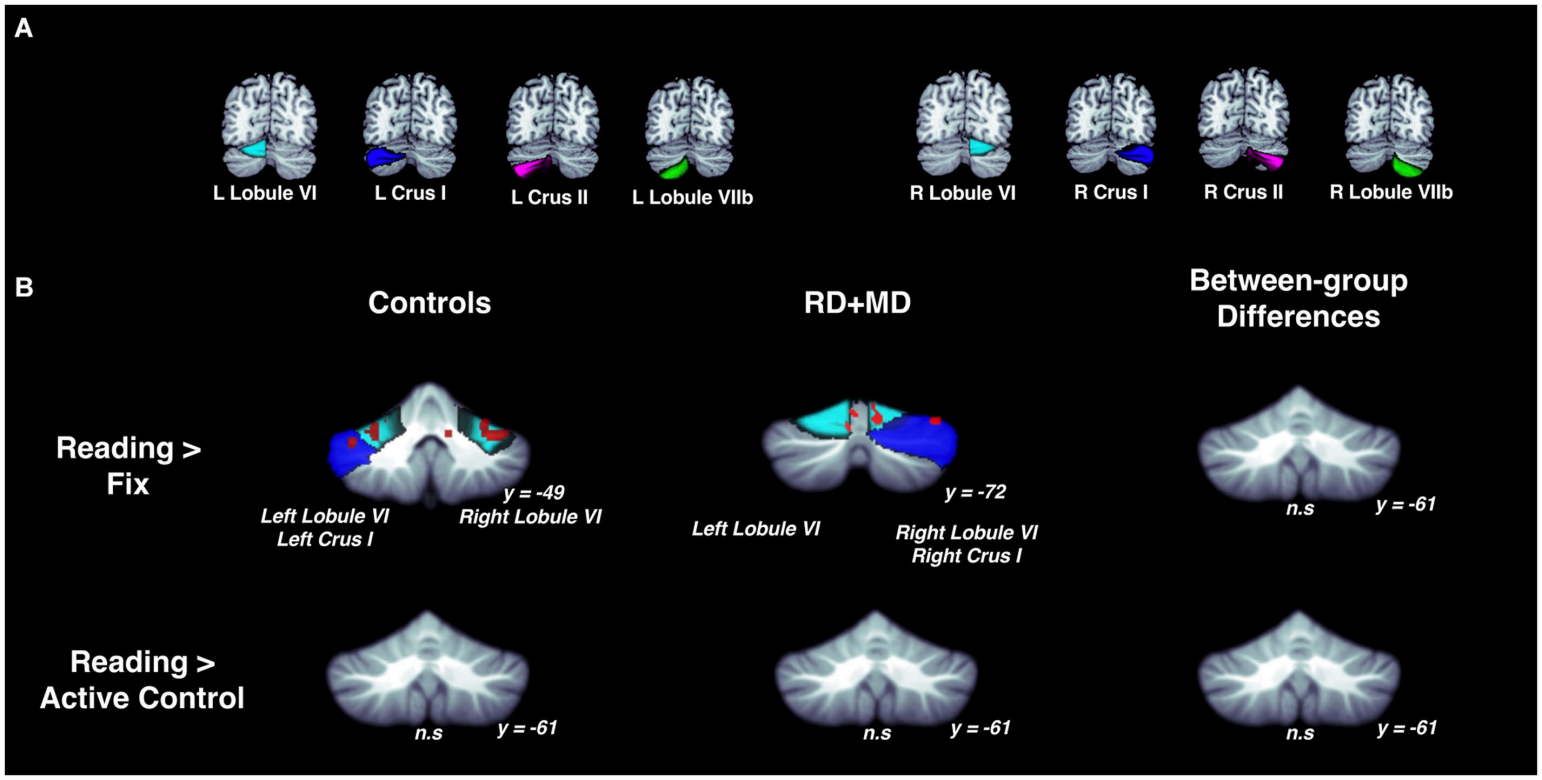
Activation during reading in Control and RD + MD groups using eight cerebellar sub-regions. ROIs included: bilateral lobule VI, crus I, crus II, lobule VIIb. **(A)** Location of the eight cerebellar sub-regions. **(B)** Reading > Fixation and Reading > Active Control contrasts. Significant activation in bilateral lobule VI and bilateral crus I for Reading > Fixation in Controls. Also, significant activation in left lobule VI, right lobule VI, and right crus I. Height threshold *p* < 0.001, *p*-FWE < 0.05 and Bonferroni-corrected so that significance was *p* < 0.00625. No significant activation for Reading > Active Control in Controls, RD + MD, nor between-group differences. Corresponding coordinates in Table 2.

For the Control group, within-group maps at the level of the whole cerebellum for the Reading task contrasted to the low-level Fixation task revealed vermis VI, left crus I, and right lobule VI. However, there were no results when contrasting the Reading task with the Active Control task, indicating no activity specific to reading (Figure 3; Table 1). Next, at the level of the eight cerebellar sub-regions, the Reading task contrasted to the low-level Fixation task revealed left lobule VI, left crus I and right lobule VI. However, there were again no results when contrasting the Reading with the Active Control task (Figure 4; Table 2), as reported in Ashburn et al. (2020).

**Table 1:** Functional activation results for whole cerebellum analysis for control and RD + MD groups during word processing in Study 1.

		MNI Coordinates			Volume		
Group	Contrast	*x*	*y*	*z*	(voxels)	*p*-value	Anatomical region
Control							
	*Reading > Fix*	−2	−76	−16	500	<0.001	Vermis VI
		−50	−56	−32	268	<0.001	Left Crus I
		32	−52	−28	200	<0.001	Right Lobule VI
	*Reading > Active Control*	*none*					
RD + MD							
	*Reading > Fix*	−2	−68	−18	259	<0.001	Vermis VI
		46	−58	−30	117	0.001	Right Crus I
		28	−58	−48	49	0.039	Right Lobule VIIIa
	*Reading > Active Control*	*none*					
Control > RD + MD							
	*Reading > Fix*	*none*					
	*Reading > Active Control*	*none*					
RD + MD > Control							
	*Reading > Fix*	*none*					
	*Reading > Active Control*	*none*					

Significance was determined by height threshold = 0.001, *p* < 0.05 FWE-corrected. *p*-values for all significant findings are listed. ‘none’ indicates no significant findings for group and/or between-group comparisons.

**Table 2:** Functional activation for cerebellar sub-region analyses for control and RD + MD children during word processing in Study 1.

			MNI Coordinates			Volume	
Group	Cerebellar Sub-regions	Contrast	*x*	*y*	*z*	(voxels)	*p*-value
Control							
	*L Lobule VI*	*RW > Fix*	−32	−38	−26	143	<0.001
			−2	−76	−18	120	<0.001
		*RW > FF*					*n.s.*
	*R Lobule VI*	*RW > Fix*	32	−52	−28	189	<0.001
		*RW > FF*					*n.s.*
	*L Crus I*	*RW > Fix*	−50	−56	−32	151	<0.001
		*RW > FF*					*n.s.*
	*All other sub-regions*	*RW > Fix*					*n.s.*
		*RW > FF*					*n.s.*
RD + MD							
	*L Lobule VI*	*RW > Fix*	−2	−64	−18	76	*0.001*
		*RW > FF*					*n.s.*
	*R Lobule VI*	*RW > Fix*	10	−72	−22	81	*<0.001*
		*RW > FF*					*n.s.*
	*R Crus I*	*RW > Fix*	46	−58	−30	108	*<0.001*
		*RW > FF*					*n.s.*
	*All other sub-regions*	*RW > Fix*					*n.s.*
		*RW > FF*					*n.s.*
Control > RD + MD							
	*All sub-regions*	*RW > Fix*					*n.s.*
		*RW > FF*					*n.s.*
RD + MD > Control							
	*All sub-regions*	*RW > Fix*					*n.s.*
		*RW > FF*					*n.s.*

Significance was determined by height-threshold <0.001, *p*-FWE <0.05 and Bonferroni-corrected for the comparison of multiple cerebellar sub-regions. *p*-values for all significant findings are listed. ‘n.s.’ indicated no significant findings.

##### 3.1.2.2 RD + MD group

For the RD + MD group, the whole-cerebellum analysis for the Reading task contrasted to Fixation revealed vermis VI, right crus I, and right lobule VIIIa. However, there were no results when contrasting Reading to the Active Control task (Figure 3; Table 1). Analysis of cerebellar sub-regions for the Reading task contrasted to Fixation also revealed right crus I, as well as left lobule VI and right lobule VI. Yet again, there were no results for these sub-regions when comparing Reading to the Active Control task (Figure 4; Table 2).

##### 3.1.2.3 Differences between control and RD + MD groups

There were no findings of activation differences between the Control and RD + MD groups for the Reading task using either comparison (Fixation or Active Control tasks), neither at the whole-cerebellum (Figure 3; Table 1) nor at the cerebellar sub-region level of analysis (Figure 4; Table 2). Bayesian analyses for these cerebellar sub-regions confirmed the results from the frequentist analyses, revealing evidence for the null hypothesis in all ROIs, with no regions showing evidence for the alternative hypothesis. For the Reading task in comparison to Fixation, BF01 values ranged from 1.3 to 3.5. “Substantial” evidence (BF > 3) for the null hypothesis (Wetzels et al., 2011; Kelter, 2020) was found in five of the eight sub-regions: left crus I and lobule VIIb, as well as right crus I, crus II, and lobule VIIb. Values were similar for the Reading task in comparison to the Active Control task (BF01 values ranged from 1.9 to 3.5), with these same five regions and also left crus II revealing “substantial” evidence (BF > 3) for the null hypothesis. As such, for the majority of cerebellar sub-regions there was more than three times the evidence for the null hypothesis (BF > 3) than the alternative hypothesis when comparing the Control and RD + MD groups, in support of no between-group differences.

#### 3.1.3 Background functional connectivity of the cerebellum with cortical reading-related regions

To test for FC independent of word processing, we performed background FC analyses of predetermined cerebellar seed and cortical target regions. Significance was determined by seed-level correction, p-FDR < 0.05. Positive *t*-statistics represent positive connectivity and negative *t*-statistics represent negative connectivity.

##### 3.1.3.1 Control group

**Figure 5:**
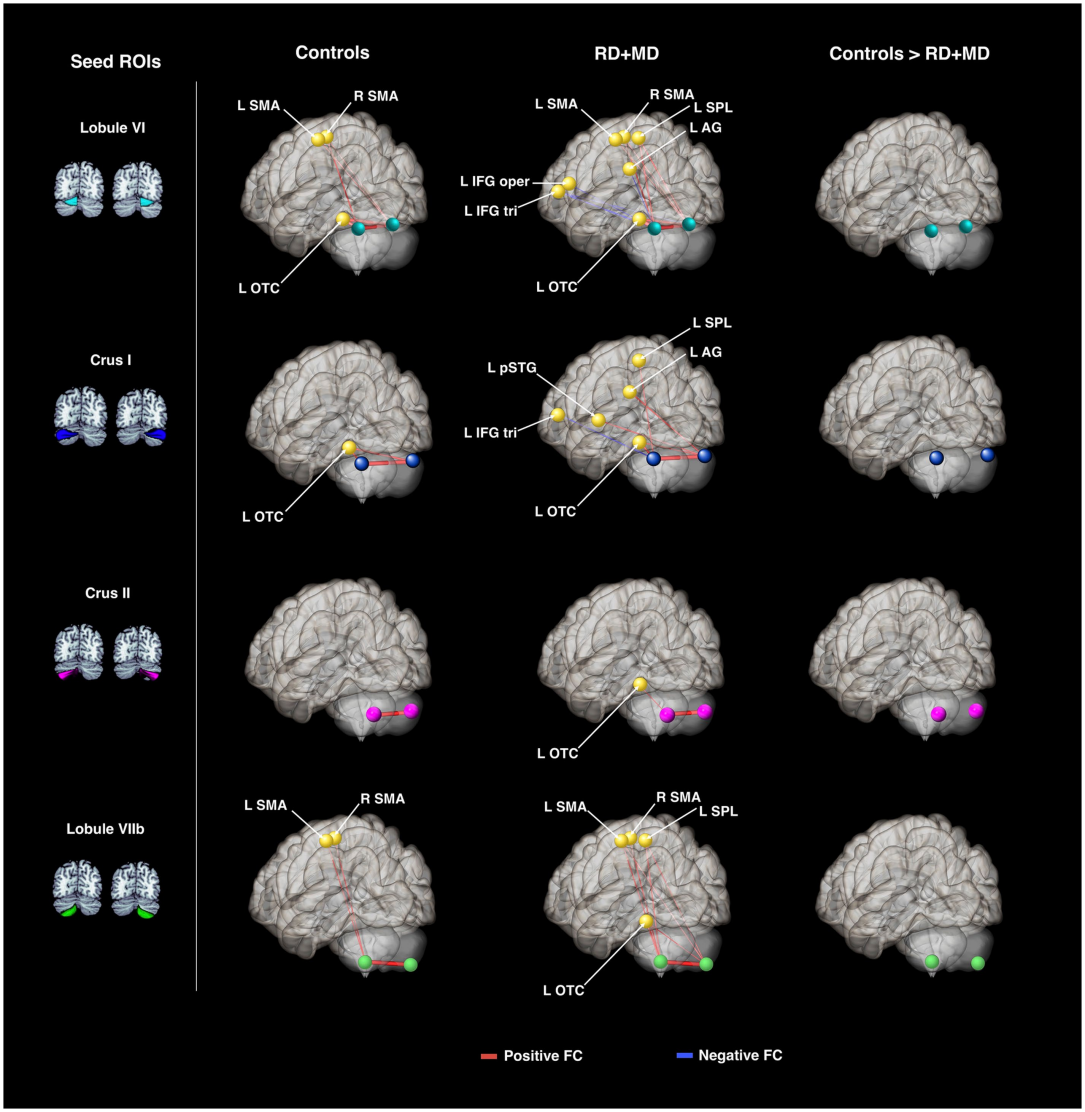
Cerebellar background functional connectivity in Control and RD + MD groups with cortical reading-related target regions. Left and right cerebellar seed regions: lobule VI, crus I, crus II, and lobule VIIb. In Controls, FC was largely limited to within the cerebellum, and between the cerebellum and left occipital-temporal cortex. RD + MD group had FC from left and right cerebellar seed regions to several left hemisphere cortical regions, including posterior superior temporal gyrus and superior parietal lobule. No significant differences between the two groups. All results corrected for multiple comparisons, *p*-FDR < 0.05, two-sided statistic.

In the Control group, every seed region exhibited positive background FC with at least one structure. Specifically, left lobule VI showed positive FC with right lobule VI, left occipital temporal cortex, and right SMA. Right lobule VI had positive FC with left lobule VI, left occipital temporal cortex, left SMA, and right SMA. Left crus I had positive FC with right crus I and left occipital temporal cortex. Right crus I had positive FC with left crus I and occipital temporal cortex. Left crus II only showed positive FC with right crus II and vice versa. Left lobule VIIb showed positive FC with right lobule VIIb, left SMA, and right SMA. Lastly, right lobule VIIb only had positive FC with left lobule VIIb (Figure 5; Table 3). These results in typical children were reported in Ashburn et al. (2020) with the current analysis also including the left and right SMA.

**Table 3:** Cerebellar background functional connectivity with cortical reading-related target regions in control and RD + MD groups in Study 1.

	Control		RD + MD		Control > RD + MD	
Seed region	FC with…	T(22)	FC with…	T(25)	FC with…	T(47)
Left lobule VI	R Lobule VI	12.22	R Lobule VI	14.58		
	L OTC	8.43	L OTC	13.65		
			L SPL	4.20		
			L AG	−2.30		
	R SMA	3.42	R SMA	3.53		
			L IFG oper	−2.46		
			L IFG tri	−4.95		
Right lobule VI	L Lobule VI	12.22	L Lobule VI	14.58		
	L OTC	9.56	L OTC	8.5		
			L SPL	4.53		
	R SMA	2.60	R SMA	4.03		
	L SMA	3.42	L SMA	3.43		
			L IFG tri	−2.84		
Left crus I	R Crus I	9.52	R Crus I	11.84		
	L OTC	3.45	L OTC	5.25		
			L SPL	2.54		
			L IFG tri	−3.01		
Right crus I	L Crus I	9.52	L Crus I	11.84		
	L OTC	4.05	L OTC	2.82		
			L AG	3.56		
			L pSTG	3.11		
Left crus II	R Crus II	12.49	R Crus II	17.60		
			L OTC	3.11		
Right crus II	L Crus II	12.49	L Crus II	17.60		
				
Left lobule VIIb	R Lobule VIIb	14.03	R Lobule VIIb	15.42		
			L OTC	3.53		
			L SPL	3.55		
	R SMA	2.95	R SMA	3.45		
	L SMA	2.78	L SMA	3.49		
Right lobule VIIb	L Lobule VIIb	14.03	L Lobule VIIb	15.42		
			L OTC	2.86		
			L SPL	2.79		
			L SMA	4.10		

Significance was determined by seed-level correction, *p*-FDR < 0.05. Positive *t*-statistics represent positive connectivity and negative *t*-statistics represent negative connectivity.

##### 3.1.3.2 RD + MD group

In the RD + MD group, again every seed region had positive background FC with at least one other structure and some had negative background FC. Specifically, left lobule VI had positive FC with right lobule VI, left occipital temporal cortex, left superior parietal lobule, and right SMA; as well as negative FC with left angular gyrus, inferior frontal gyrus *pars opercularis,* and inferior frontal gyrus *pars triangularis*. Right lobule VI showed positive FC with left lobule VI, occipital temporal cortex, superior parietal lobule, SMA, and right SMA, as well as negative FC with left inferior frontal gyrus *pars triangularis*. Left crus I had positive FC with right crus I, left occipital temporal cortex, superior parietal lobule, as well as negative FC with left inferior frontal gyrus *pars triangularis*. Right crus I showed positive FC with left crus I, occipital temporal cortex, angular gyrus, and posterior superior temporal gyrus. Left crus II had positive FC with right crus II and left occipital temporal cortex. Right crus II only had positive FC with left crus II. Left lobule VIIb had positive FC with left occipital temporal cortex, superior parietal lobule, SMA, and right SMA. Lastly, right lobule VIIb had positive FC with left lobule VIIb, occipital temporal cortex, superior parietal lobule, and supplementary motor area (Figure 5; Table 3).

##### 3.1.3.3 Differences between control and RD + MD groups

No significant differences emerged from the comparison between the Control and RD + MD groups on background connectivity (Figure 5; Table 3).

#### 3.1.4 Task-dependent functional connectivity of the cerebellum with cortical reading-related regions

To test for FC during word processing, we performed gPPI analyses of our predetermined cerebellar seed and cortical target regions. Significance was determined by seed-level correction, p-FDR < 0.05. Positive *t*-statistics represent positive connectivity and negative *t*-statistics represent negative connectivity.

##### 3.1.4.1 Control group

**Figure 6:**
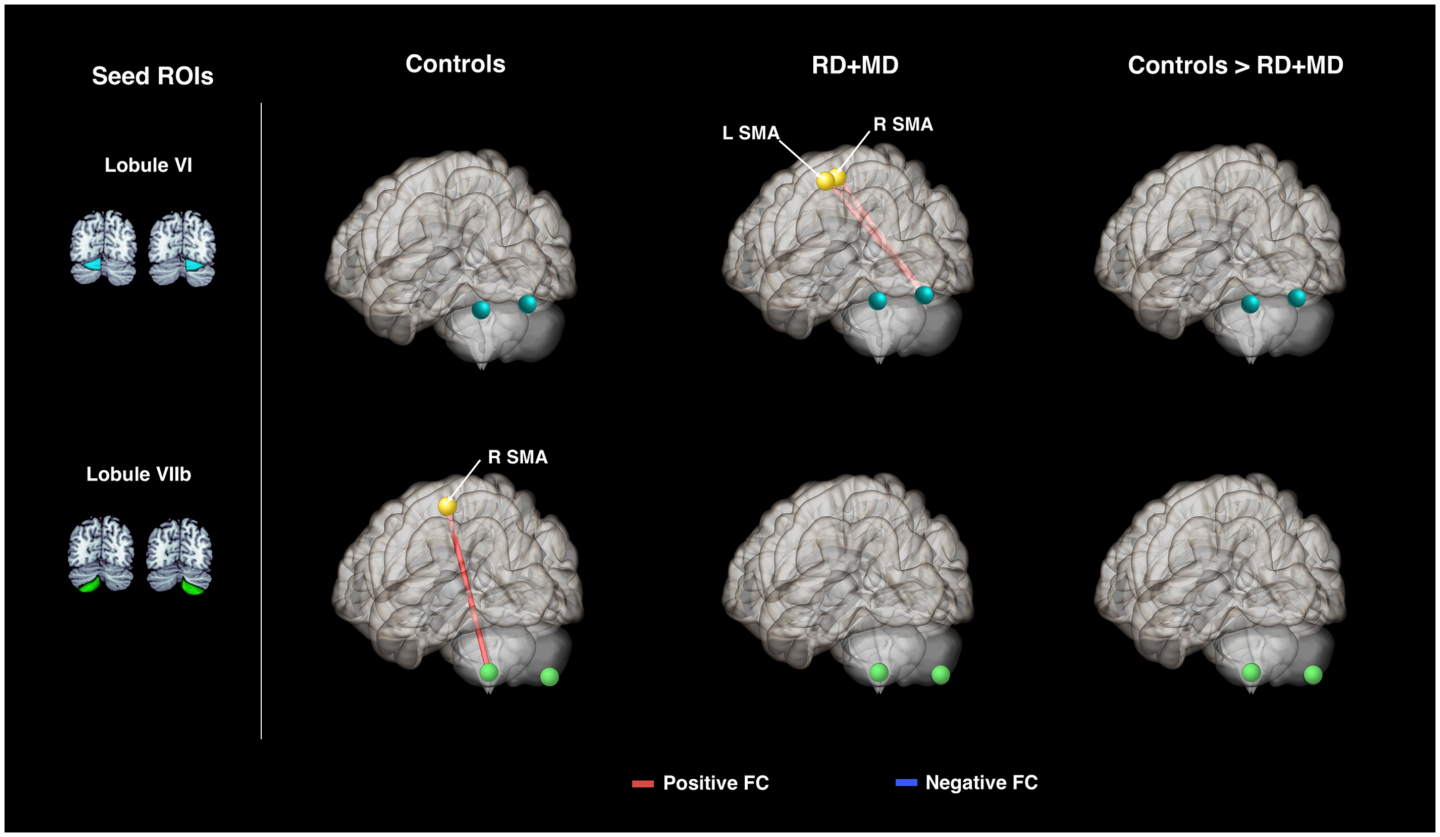
Cerebellar task-dependent functional connectivity in Control and RD + MD groups with cortical reading-related target regions. All eight cerebellar seeds were tested; however only those with findings are displayed here. In the Control group, left lobule VIIb had positive task-dependent FC with right supplementary motor area during single word processing. In RD + MD group, right lobule VI had positive task-dependent FC with left and right supplementary motor area during word processing.

The Control group had a FC connection modulated by word processing between left lobule VIIb and right SMA (Figure 6; Table 4).

**Table 4:** Cerebellar task-dependent functional connectivity with cortical reading-related target regions in control and RD + MD children in Study 1.

	Control		RD+MD		Control > RD+MD	
Seed region	FC with…	T(22)	FC with…	T(25)	FC with…	T(47)
Right lobule VI			L SMA	2.96		
			R SMA	3.45		
Left lobule VIIb	R SMA	3.23				
All other cerebellar seeds		n.s.		n.s.		

Significance was determined by seed-level correction, *p*-FDR < 0.05. Positive *t*-statistics represent positive connectivity and negative *t*-statistics represent negative connectivity. Only cerebellar seeds with significant results are shown.

##### 3.1.4.2 RD + MD group

In the RD + MD group, only one cerebellar seed region had significant FC that was modulated by word processing. Specifically, right lobule VI had positive FC with left and right SMA (Figure 6; Table 4).

##### 3.1.4.3 Differences between control and RD + MD groups

No significant differences emerged when comparing between the Control and RD + MD groups for FC specific to reading (Figure 6; Table 4).

### 3.2 Study 2: arithmetic processing

#### 3.2.1 Behavioral measures

Accuracy and response time for Control group and RD + MD group are shown in Supplementary Table 4. Most relevant to our fMRI activation analyses is that there were no significant differences between the Control group and RD + MD group for accuracy or response time when comparing the difference between the Arithmetic and the Active Control task. As in Study 1, this is important since this is the contrast used for the activation analysis to identify areas specific to arithmetic processing.

#### 3.2.2 Arithmetic processing: activation analysis constrained to (i) the whole cerebellum and (ii) cerebellar sub-regions (left and right lobule VI, crus I, crus II, lobule VIIb)

The reporting of significant results for both within- and between-groups are based on a height threshold = 0.001, *p* < 0.05 FWE-corrected (for whole cerebellum) and *p* < 0.00625 FWE-Bonferroni-corrected (for cerebellar sub-regions).

##### 3.2.2.1 Control group

**Figure 7:**
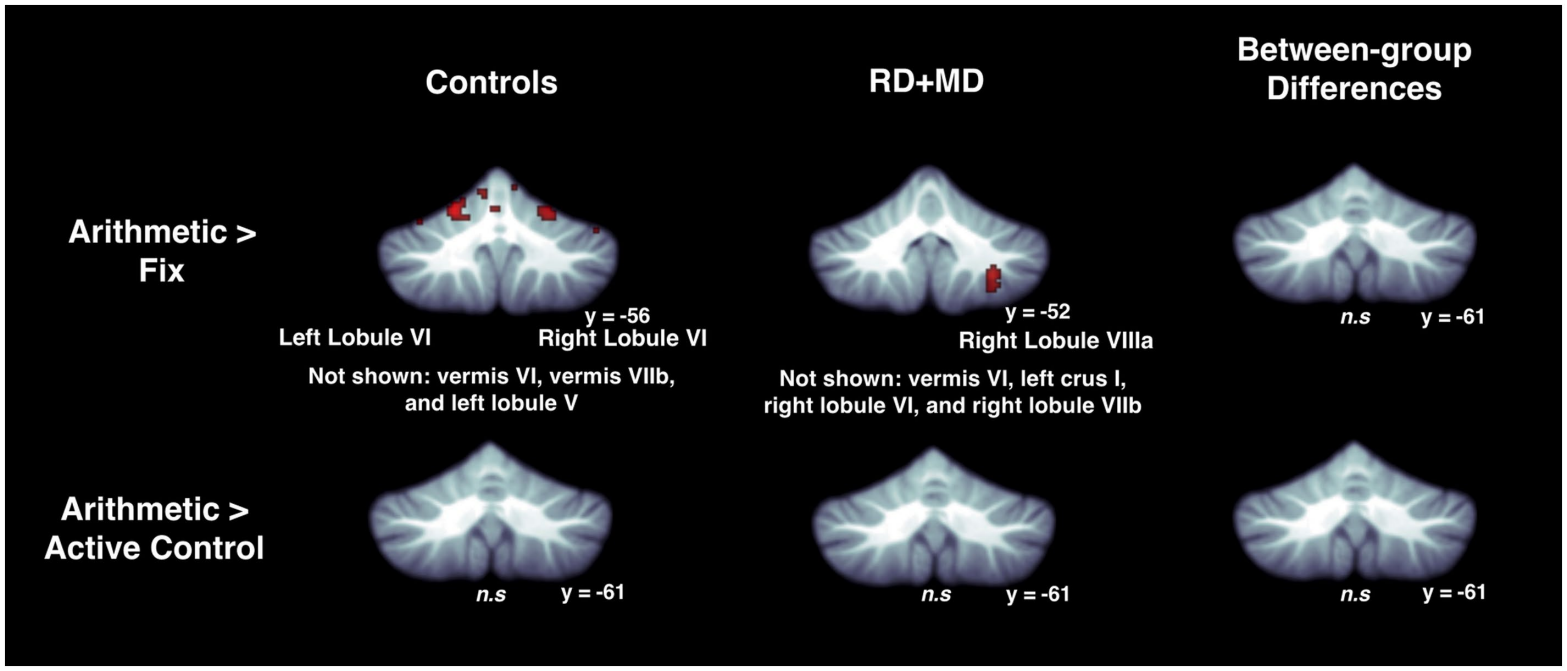
Activation during arithmetic in Control and RD + MD groups using whole cerebellum analyses. Arithmetic > Fixation contrast, and Arithmetic > Active Control contrast. Significant activation in left lobule VI and right lobule VI, as well as regions not shown including vermis VI, vermis VIIb, and left lobule V in the Control group, height threshold *p* < 0.001, *p* < 0.05 FWE-corrected. Also, activation in right lobule VIIIa, as well as regions not shown including, vermis VI, left crus I, right lobule VI, and right lobule VIIb in the RD + MD group. No significant activation for Arithmetic > Active Control for either group and no between-group differences for either contrast.

**Figure 8:**
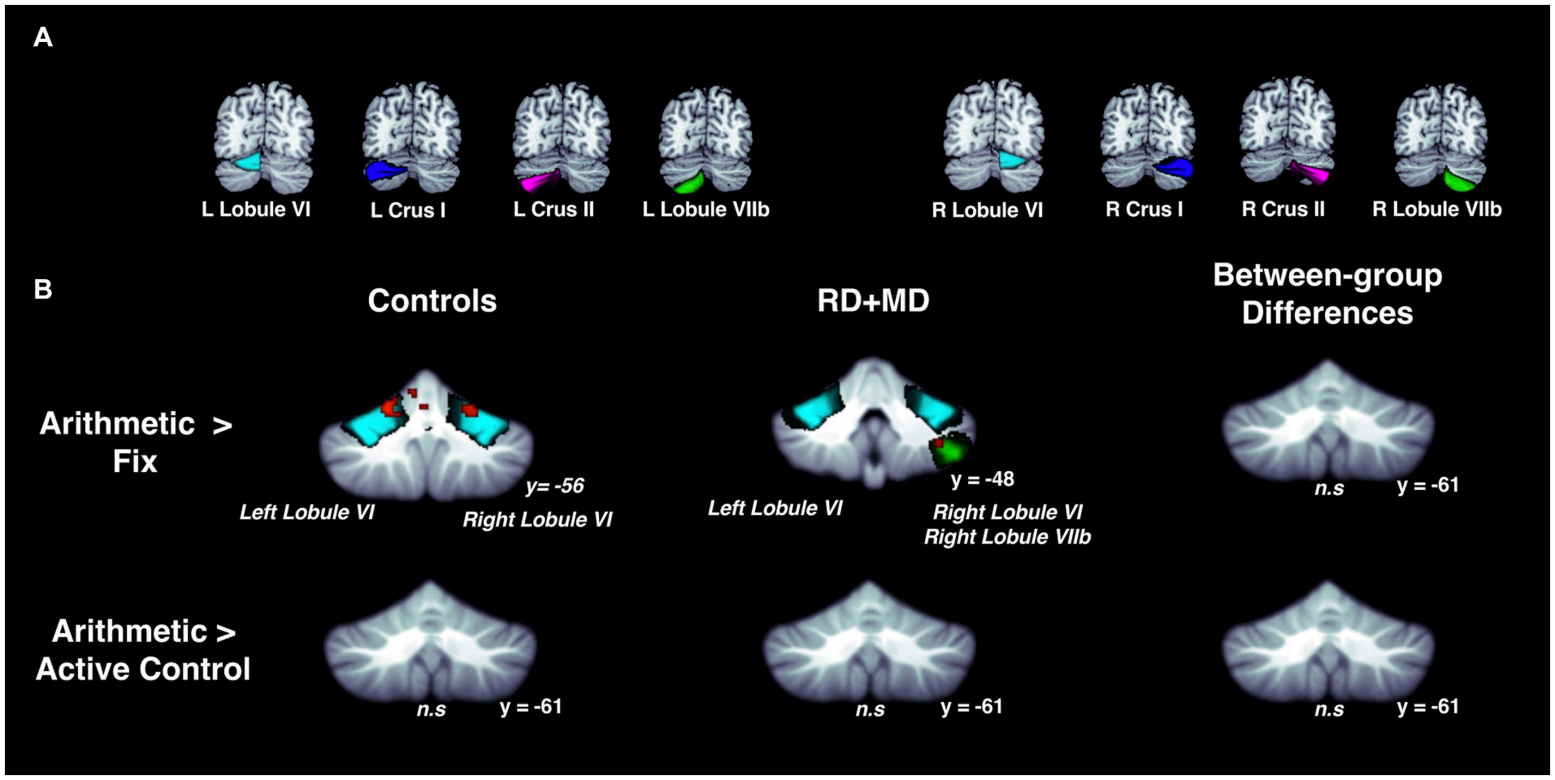
Activation during arithmetic in Control and RD + MD groups using eight cerebellar sub-regions. ROIs included: bilateral lobule VI, crus I, crus II, and lobule VIIb. **(A)** Location of the eight cerebellar sub-regions. **(B)** Arithmetic > Fixation contrast, and Arithmetic > Active Control contrast. Significant activation in left and right lobule VI in Control group, height threshold *p* < 0.001, *p* < 0.05 FWE-corrected. Also, there was activation in left lobule VI, right lobule VI, and right lobule VIIb in the RD + MD group. No significant activation for Arithmetic > Activate Control for either group and no between-group differences for either contrast.

For the Control group, within-group maps at the level of the whole cerebellum for Arithmetic contrasted to Fixation revealed vermis VI and vermis VIIb, left lobule V and lobule VI, and right lobule VI. However, there were no results when contrasting Arithmetic with the Active Control condition (Figure 7; Table 5). Next, at the level of the eight cerebellar sub-regions, Arithmetic contrasted to Fixation revealed left and right lobule VI. However, there again were no results when contrasting Arithmetic to the Active Control task (Figure 8; Table 6).

**Table 5:** Functional activation results for whole cerebellum analysis for control and RD + MD groups during arithmetic processing in Study 2.

		MNI coordinates			Volume		
Group	Contrast	*x*	*y*	*z*	(voxels)	*p*-value	Anatomical region
Control							
	*Arithmetic > Fixation*	−4	−80	−24	59	0.018	Vermis VI
		6	−70	−30	61	0.015	Vermis VIIb
		0	−60	−18	92	0.002	Left Lobule V
		−20	−56	−18	93	0.002	Left Lobule VI
		22	−64	−14	109	0.001	Right Lobule VI
	*Arithmetic > Active Control*	*none*					
RD + MD							
	*Arithmetic > Fixation*	−4	−72	−16	281	<0.001	Vermis VI
		−38	−60	−32	38	0.036	Left Crus I
		34	−48	−28	39	0.033	Right Lobule VI
		42	−60	−52	35	0.046	Right Lobule VIIb
		28	−54	−48	97	0.001	Right Lobule VIIIa
	*Arithmetic > Active Control*	*none*					
Control > RD + MD							
	*Arithmetic > Fixation*	*none*					
	*Arithmetic > Active Control*	*none*					
RD + MD > Control							
	*Arithmetic > Fixation*	*none*					
	*Arithmetic > Active Control*	*none*					

Significance was determined by height threshold = 0.001, *p* < 0.05 FWE-corrected. *p*-values for all significant findings are listed. ‘none’ indicates no significant findings for group and/or between-group comparison.

**Table 6:** Functional activation for cerebellar sub-region analyses for control and RD + MD children during arithmetic processing in Study 2.

			MNI coordinates			Volume	
Group	Cerebellar sub-regions	Contrast	*x*	*y*	*z*	(voxels)	*p* value
Control							
	*L Lobule VI*	*Arithmetic > Fix*	−4	−80	−24	51	0.003
			−20	−56	−18	61	0.001
		*Arithmetic > Active Control*					*n.s.*
	*R Lobule VI*	*Arithmetic > Fix*	22	−64	−14	74	0.001
		*Arithmetic > Active Control*					*n.s.*
	*All other sub-regions*	*Arithmetic > Fix*					*n.s.*
		*Arithmetic > Active Control*					*n.s.*
RD + MD							
	*L Lobule VI*	*Arithmetic > Fix*	−4	−72	−16	116	<0.001
		*Arithmetic > Active Control*					*n.s.*
	*R Lobule VI*	*Arithmetic > Fix*	8	−70	−14	85	<0.001
		*Arithmetic > Active Control*					*n.s.*
	*R Lobule VIIb*	*Arithmetic > Fix*	30	−60	−44	44	0.003
		*Arithmetic > Active Control*					*n.s.*
	*All other sub-regions*	*Arithmetic > Fix*					*n.s.*
		*Arithmetic > Active Control*					*n.s.*
Control > RD + MD							
	*All sub-regions*	*Arithmetic > Fix*					*n.s.*
		*Arithmetic > Active Control*					*n.s.*
RD + MD > Control							
	*All sub-regions*	*Arithmetic > Fix*					*n.s.*
		*Arithmetic > Active Control*					*n.s.*

Significance was determined by height-threshold <0.001, *p*-FWE <0.05 and Bonferroni-corrected for the comparison of multiple cerebellar sub-regions. *p* values for all significant findings are listed. ‘n.s.’ indicated no significant findings.

##### 3.2.2.2 RD + MD group

For the RD + MD group, the whole-cerebellum analysis for Arithmetic contrasted to Fixation revealed vermis VI, left crus I, as well as right lobule VI, lobule VIIb, and lobule VIIIa. However, there were no results when contrasting Arithmetic to the Active Control task (Figure 7; Table 5). The cerebellar sub-region analysis contrasting Arithmetic to Fixation revealed activation in left lobule VI, and right lobule VI and lobule VIIb. However, there were no results when contrasting Arithmetic with the Active Control task (Figure 8; Table 6).

##### 3.2.2.3 Differences between control and RD + MD groups

There were no findings of activation differences between the between the Control and RD + MD groups for the Arithmetic task using either comparison (Fixation or Active Control tasks), neither at the whole-cerebellum (Figure 7; Table 5) nor cerebellar sub-region level of analysis (Figure 8; Table 6). Bayesian analyses for these cerebellar sub-regions was consistent with the results from the frequentist analyses, revealing evidence for the null hypothesis in all ROIs, with no regions showing evidence for the alternative hypothesis. For the Arithmetic task in comparison to Fixation, BF01 values ranged from 2.1 to 2.9. Values were similar for the Arithmetic task in comparison to the Active Control task (BF01 values ranged from 1.6 to 3.4), this time with right Crus I revealing “substantial” evidence (BF > 3) for the null hypothesis. As such, for all of the cerebellar sub-regions there was more than two or three times the evidence for the null model than the alternative hypothesis when comparing the Control and RD + MD groups, indicative of an absence of evidence for between-group differences.

#### 3.2.3 Background functional connectivity of the cerebellum with cortical math-related regions

To test for intrinsic FC (independent of arithmetic processing), we performed background FC analyses of predetermined cerebellar seed and cortical target regions. As in Study 1, significance was determined by seed-level correction, p-FDR < 0.05. Positive t-statistics represent positive connectivity and negative *t*-statistics represent negative connectivity.

##### 3.2.3.1 Control group

**Figure 9:**
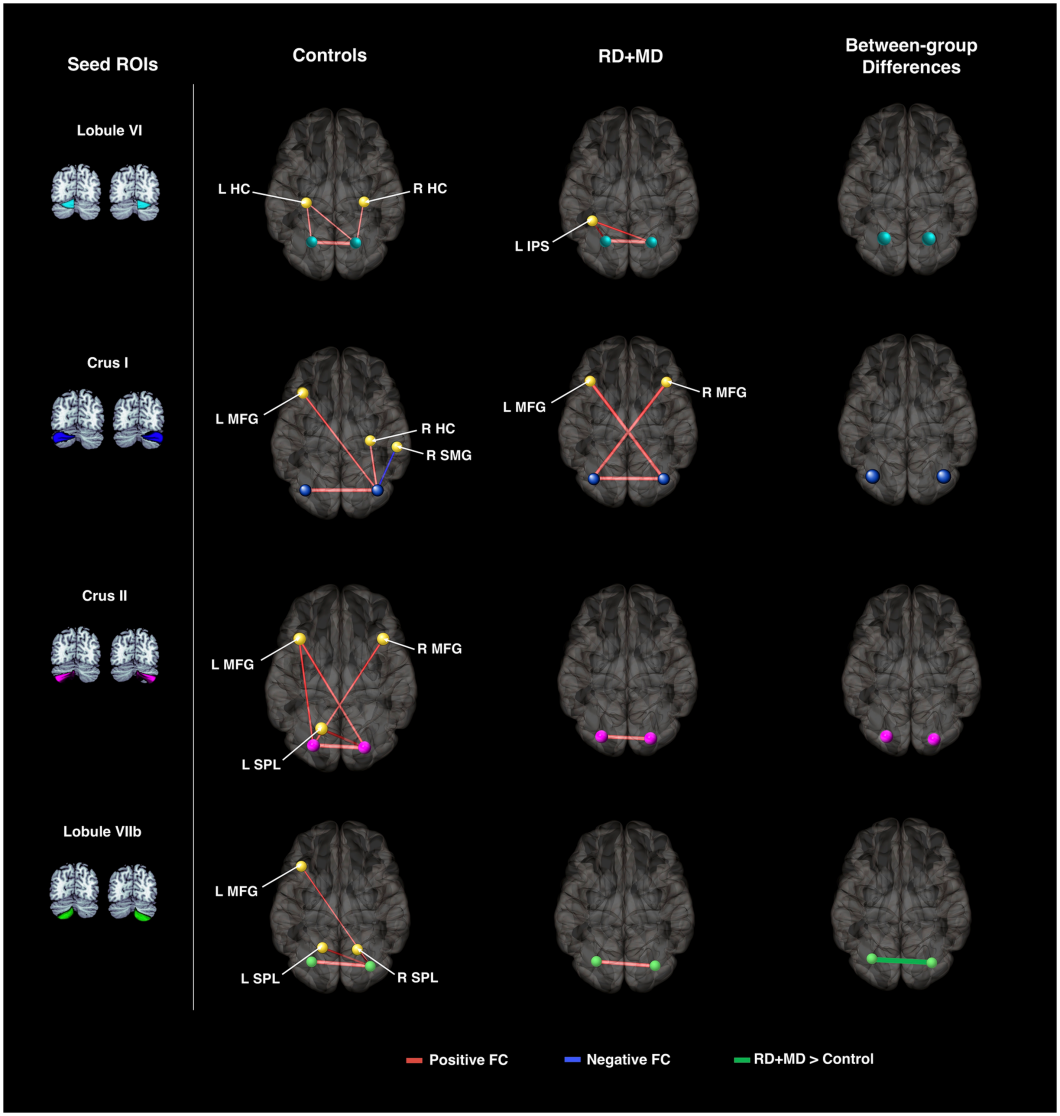
Cerebellar background functional connectivity in Control and RD + MD groups with cortical math-related target regions. Left and right cerebellar seed regions: lobule VI, crus I, crus II, and lobule VIIb with math-related cortical target regions. Brains are oriented from a superior view. In Controls, FC of cerebellar seed regions was largely with bilateral hippocampal cortex, middle frontal gyrus, and superior parietal lobule. RD + MD group had FC from left and right lobule VI with left intraparietal sulcus as well as left and right crus I with the contralateral middle frontal gyrus for each. No significant differences between the two groups for cerebellar FC with cortical target regions, but FC between left and right lobule VIIb was greater for the RD+MD group than the Control group. All results corrected for multiple comparisons, *p*-FDR < 0.05, two-sided statistic.

In the Control group, every seed region exhibited positive background FC with at least one region and one had negative background FC. Left lobule VI had positive FC with right lobule VI and left hippocampus. Right lobule VI had positive FC with left lobule VI, left hippocampus, and right hippocampus. Left crus I only had positive FC with right crus I. Right crus I had positive FC with left crus I, left middle frontal gyrus, and right hippocampus as well as negative FC with right supramarginal gyrus. Left crus II had positive FC with right crus II and middle frontal gyrus as well as left middle frontal gyrus. Right crus II had positive FC with left crus II, middle frontal gyrus, and superior parietal lobule. Left lobule VIIb only had FC with right lobule VIIb. Right lobule VIIb had positive FC with left lobule VIIb, middle frontal gyrus, superior parietal lobule, and right superior parietal lobule (Figure 9; Table 7).

**Table 7:** Cerebellar background functional connectivity with cortical math-related target regions in control and RD + MD children in Study 2.

	Control		RD + MD		Control > RD + MD	
Seed region	FC with…	T(15)	FC with…	T(13)	FC with…	T(28)
Left lobule VI	R Lobule VI	7.29	R Lobule VI	8.01		
	L HC	3.29				
			L IPS	3.72		
Right lobule VI	L Lobule VI	7.29	L Lobule VI	8.01		
	L HC	3.28				
	R HC	3.12				
			L IPS	4.12		
Left crus I	R Crus I	5.85	R Crus I	5.06		
			R MFG	4.14		
Right crus I	L Crus I	5.85	L Crus I	5.06		
	R HC	3.22				
	L MFG	3.21	L MFG	4.55		
	R SMG	−3.19				
Left crus II	R Crus II	6.40	R Crus II	11.44		
	L MFG	3.18				
	R MFG	3.21				
Right crus II	L Crus II	6.40	L Crus II	11.44		
	L MFG	3.65				
	L SPL	4.23				
Left lobule VIIb	R Lobule VIIb	7.77	R Lobule VIIb	22.73	R Lobule VIIb	−3.39
Right lobule VIIb	L Lobule VIIb	7.77	L Lobule VIIb	22.73	L Lobule VIIb	−3.39
	L MFG	2.96				
	L SPL	4.32				
	R SPL	2.87				

Significance was determined by seed-level correction, *p*-FDR < 0.05. Positive *t*-statistics represent positive connectivity and negative *t*-statistics represent negative connectivity.

##### 3.2.3.2 RD + MD group

In the RD + MD group, again every seed region had positive background FC with at least one other region. In RD + MD group, left lobule VI had positive FC with right lobule VI and left intraparietal sulcus. Right lobule VI had positive FC with left lobule VI and left intraparietal sulcus. Left Crus I had positive FC with right crus I and right middle frontal gyrus. Right crus I had positive FC with left crus I and left middle frontal gyrus. Left crus II only had positive FC with right crus II and vice versa. Similarly, left lobule VIIb only had positive FC with right lobule VIIb and vice versa (Figure 9; Table 7).

##### 3.2.3.3 Differences between control and RD + MD groups

When testing for differences between the Control group and the RD + MD group in background FC, we found that the RD + MD group had more positive FC compared to the Control group between two homotopic regions of the cerebellum (left and right lobule VIIb), however, there were no differences for cerebellar-cortical connections (Figure 9; Table 7).

#### 3.2.4 Task-dependent functional connectivity of the cerebellum with cortical math-related regions

To test for FC during arithmetic processing, we performed gPPI analyses of our predetermined cerebellar seed and cortical target regions.

##### 3.2.4.1 Control group

The analysis from the Control group yielded no significant results.

##### 3.2.4.2 RD + MD group

The analysis from the RD + MD group no significant results.

##### 3.2.4.3 Differences between control and RD + MD groups

No significant differences emerged when comparing between the Control and RD + MD groups for FC specific to arithmetic.

### 3.3 Summary of results

For Study 1 and 2, there was activity in the cerebellum for the Control and the RD + MD groups during word processing and during arithmetic processing relative to a low-level baseline comparison condition (Fixation). However, there was no significant activation for either group specific to reading or arithmetic (i.e., when contrasting the reading or arithmetic task to the respective active control conditions). Importantly, there were no differences when comparing between the Control and RD + MD groups on activation for reading or for arithmetic (using either baseline comparison, and for whole-cerebellum and for cerebellar sub-region analyses) and these were largely supported with Bayesian analyses. For functional connectivity, in both Study 1 and Study 2 there were many incidences of background FC in both the Control and RD + MD groups between the cerebellar seed regions and cortical (reading- or math-related) target regions. However, there again were no differences between the Control and RD + MD groups for cerebellar-cortical connections. For task-dependent functional connectivity for reading in Study 1, there was one within-group result in the Controls (left lobule VIIb with right SMA), and two functional connections in the RD + MD group (right lobule VI with left and right SMA). However, again no between-group differences. For Study 2, there was no task-dependent FC during arithmetic task in the Control nor the RD + MD group and no between-group differences. Overall, the Control and the RD + MD groups did not differ in terms of activation or functional connectivity between the cerebellum and the target cortical regions in these studies of reading and arithmetic.

## 4 Discussion

We conducted studies to test the cerebellum’s involvement in word processing and arithmetic processing in children with co-occurring reading and math disabilities compared to a control group. Based on theories proposing a role of the cerebellum and its cortical connections in reading and in arithmetic (Nicolson et al., 2001; Vandervert, 2017; Nicolson and Fawcett, 2019), one might expect to find differences in activation and in functional connectivity between the group with RD + MD and the Control group. However, few prior brain imaging studies have reported differences in the cerebellum in children with RD or MD, and here we did not find such differences in children with RD + MD. Below we offer context for these results using the prior literature, which for reading and arithmetic and their disorders has focused on the cerebellum only infrequently, and instead focused on a left-hemisphere cortical network as the neural bases of reading (and RD) and a bilateral fronto-parietal network for arithmetic (and MD).

### 4.1 Functional activation of the cerebellum during word processing

As noted in the Introduction, most studies on the neural bases of reading in typically-developing children and adolescents of alphabetic languages do not report activation in the cerebellum. This was reflected in the meta-analysis results from Martin et al. (2015), which did not find convergence of activation in the cerebellum for studies of reading in children. While both the Control and the RD + MD groups in the present study activated bilateral cerebellar regions during word processing when contrasted with a low-level fixation task, this was not the case when contrasted with the active control task (false fonts). Therefore, we do not attribute this activation to reading, but to other aspects of the task (e.g., finger pressing).

In the Martin et al. (2015) meta-analysis on reading in alphabetic languages in typical children, six of the 20 original studies reported cerebellar activation (Booth et al., 2001; Gaillard et al., 2003; Hoeft et al., 2006; Noble et al., 2006; Rimrodt et al., 2009). Adding to this, a more recent study by Liebig and colleagues reported cerebellar activation of right crus I during orthographic decision, phonological decision, and semantic organization tasks, all relative to a visual line judgment baseline (Liebig et al., 2017). Our Control and RD + MD groups both activated vermis VI and left and right lobule VI during word processing compared to fixation (but not compared to active control). Right lobule VI is one region implicated in reading disability (Stoodley, 2016), but it (nor any other region of the cerebellum) did not differ in our group with RD + MD.

Here we consider our results of no differences between the group with and without RD + MD. The only study that we are aware of to have looked at word processing in children with RD + MD was in the supplementary materials of Peters et al. (2018). In that study, children made a decision on whether visually-presented words contained a specific phoneme or were presented in upper or lower case. They found no differences in activation between groups with RD + MD, RD-only, MD-only or controls at an FDR-corrected level (Peters et al., 2018). Of note, Peters and colleagues caution against making strong conclusions due to the small sample size; however, it is currently the only functional activation study of reading in children with RD + MD. This and the other activation studies described above used a whole-brain analysis approach. However, despite our use of an analysis specifically focused on the cerebellum and a larger sample size, our findings were consistent with that of Peters et al. (2018) in that activity of the cerebellum did not differ in children with and without RD + MD during word processing.

Lastly, given the focus on the cerebellum in the context of children’s poor reading skills, we turn to the only meta-analysis constrained to children comparing functional activation studies in those with and without RD in alphabetic languages. Richlan and colleagues reported convergence for relative under-activation in cortical regions known to be involved in reading in children with dyslexia (left inferior parietal lobule, supramarginal gyrus, and fusiform gyrus), but no differences in the cerebellum (Richlan et al., 2011). Only two of the nine studies included in this meta-analyses found a difference in activity in children with RD (relatively more) in the cerebellar vermis during sentence reading (Meyler et al., 2008) and letter matching (Temple et al., 2001). A study not included in the meta-analysis reported less activation in the cerebellum in RD during semantic word matching in children using an alphabetic writing system, but this result did not meet statistical significance after correcting for multiple comparisons (Hu et al., 2010). In our own previous study comparing children with and without RD, we found no differences in cerebellar activation during the same word processing task (Ashburn et al., 2020). Therefore, the findings from the current investigation are consistent with those reported in these meta-analyses and prior studies. Since activations do not provide insight into how the cerebellum may interact with cortical regions, as is implicated by the cerebellar deficit hypothesis, we went on to examine functional connectivity, as described next.

### 4.2 Functional connectivity of the cerebellum to cortical reading-related regions

We examined background functional connectivity and task-dependent functional connectivity between the cerebellum and regions known to be involved in reading. Background connectivity (Norman-Haignere et al., 2012) is comparable to resting-state connectivity, which measures intrinsic functional connectivity in the absence of a task (Fair et al., 2007). Resting-state studies in adults have shown intrinsic functional connectivity between the cerebellum and cortical regions involved in motor control, as well as regions within fronto–parietal and ventral attention networks (Buckner et al., 2011; Balsters et al., 2014; Riedel et al., 2015; Guell et al., 2018). These networks include the inferior frontal gyrus, temporal–parietal cortex, and occipital-temporal cortex, regions which are also known to be altered in reading disability (Gabrieli, 2009; Eden et al., 2015). Intrinsic functional connections have been shown to be stronger in adults than in children (Hoff et al., 2013; Grayson and Fair, 2017). Interestingly, intrinsic functional connections have been reported for children between the cerebellum and the angular gyrus as well as between the cerebellum and the intraparietal lobule (Dosenbach et al., 2010). Regions of interest are used to either conduct ROI-to-ROI or seed-to-voxel (ROI-to- the rest of the brain) analyses in studies on functional connectivity. To date, knowledge about cerebellar-cortical connections in children with learning disabilities are limited because very few of the studies on children with learning disabilities include the cerebellum as a region of interest. A cerebellar deficit (Nicolson et al., 2001) would be expected to manifest as aberrations in functional connections between cerebellar and cortical reading-related regions and this was the focus of the current study.

For background connectivity in the Control and RD + MD groups, we found that all cerebellar seed regions had at least one positive connection, and most had at least one with a cortical target (seed) region. The following connections were observed in both groups: left lobule VI with left occipital temporal cortex and right SMA; right lobule VI with left occipital temporal cortex, and left and right SMA. There was also left crus I and right crus I with left occipital temporal cortex; and left lobule VIIb with left and right SMA. These functional connections of lobules VI, crus I, and lobule VIIb with cortical regions have been associated with visual and motor processing fitting with prior studies in adults (Buckner et al., 2011; Riedel et al., 2015). While there were other connections specific to each group (including negative background connectivity), it was surprising that there were no findings of positive functional connections between cerebellar crus I and crus II with frontal language areas as would have been expected based on prior work in adults (Guell et al., 2018). There are few resting-state studies in typically developing children using alphabetic languages and some of these have used seed-to-voxel analysis focusing on regions known to be involved in reading and relating them to performance (Koyama et al., 2011, 2013; Cross et al., 2021). Of the few seed-to-voxel studies explicitly considering the cerebellum, Greeley and colleagues found a positive connection between right cerebellar lobule VIII and left angular gyrus to be related to reading scores in typical readers (Greeley et al., 2021).

When directly comparing the Control and the RD + MD group, there were no differences in background connectivity between the RD + MD group and the controls. We are aware of only one resting-state FC study which compared RD + MD children to RD-only, MD-only and Controls (Skeide et al., 2018). It reported weaker FC in RD + MD children between right para-hippocampal gyrus and left posterior fusiform gyrus in comparison to the other three groups. Of note, this was a ROI-to-ROI FC analysis and did not include the cerebellum.

Again, turning to the literature on children with only reading disability, most studies in alphabetic languages have examined intrinsic cortical FC at network-level with none reporting findings in the cerebellum (Koyama et al., 2013; Horowitz-Kraus et al., 2018; Twait et al., 2018; Freedman et al., 2020). However, the recent seed-to-voxel study by Greeley et al. (2021) noted above, also included children with RD. Of the 18 ROIs placed in the cerebellum, four showed multiple differences in connectivity between the two groups. Right crus I and lobule VI seeds had stronger as well as weaker functional connectivity with various cortical regions in the group with RD relative to controls. Right lobule VII and lobule VIII seeds only had stronger positive functional connectivity with various cortical regions in the group with RD. Further, a functional connection between right cerebellar lobule VIII and left angular gyrus was positively correlated with reading ability in the RD group and trending in the control group. In our previous study comparing children with and without dyslexia, we found more positive intrinsic FC between right crus I and cortical regions of the reading network (left angular, posterior superior temporal, and inferior frontal gyri) in children with RD than the controls (Ashburn et al., 2020). This was not found by Greeley et al. (2021) in their study of RD and also not in the current study of RD + MD. Lastly, an intrinsic functional connectivity study in dyslexia (and developmental coordination disorder) found that the group with RD was not characterized by impaired connectivity in the cortico-cerebellar network (Cignetti et al., 2020).

Moving on from these task-independent functional connections, we then used a gPPI analysis to test for functional connections associated with word processing using the same cerebellar seed and cortical target regions. This time there were few functional connections in the Control and in the RD + MD groups. In the Controls, the cerebellum’s left lobule VIIb had positive task-dependent functional connectivity with right SMA (consistent with the background connectivity finding as described above). In the RD + MD group, the cerebellum’s right lobule VI had positive task-dependent functional connectivity with left and right SMA (consistent with the background connectivity finding described above). However, there were no significant differences between the two groups.

Few studies have investigated task-dependent FC connections during reading in alphabetic writing systems in typical children (Wang et al., 2013; Morken et al., 2017) and of these none included the cerebellum as a region of interest. We are not aware of any task-dependent functional connectivity studies in children with RD + MD using a reading task in an alphabetic language. There have been studies that test for task-dependent FC in children with RD during reading (Richards and Berninger, 2008; Ashburn et al., 2020; Kim et al., 2022) and of these two included the cerebellum (Richards and Berninger, 2008; Ashburn et al., 2020). Richards and Berninger conducted a seed-to-voxel analysis during the visual presentation of a phoneme-mapping mapping task and found between-group differences in FC for the seed in left inferior frontal gyrus with other regions; however, most relevant to the present study, there were no differences in their cerebellar seed region with cortical regions (Richards and Berninger, 2008). Ashburn et al. (2020) also found no differences in task-dependent connectivity between the cerebellum and cortical reading-related regions for children with and without RD.

### 4.3 Functional activation of the cerebellum during arithmetic processing

In general, not many studies on the neural bases of arithmetic in typically developing children find the cerebellum to be activated. While both the Control and the RD + MD groups in the present study had activation in bilateral cerebellum during arithmetic processing when contrasted with a low-level fixation, this was not the case when contrasted with the active control task. As in Study 1, we conclude that this more controlled comparison, which accounts for other aspects of the task, indicates that the cerebellum is not active during arithmetic, specifically, in either group, consistent with prior studies of typical children. A meta-analysis of arithmetic processing in typically developing children found no convergence of cerebellar activation during arithmetic tasks (Arsalidou et al., 2018). Of the studies included in this meta-analysis, only seven of the 17 studies found activation in the cerebellum for various math-related tasks (Meintjes et al., 2010; de Smedt et al., 2011; Mondt et al., 2011; Ashkenazi et al., 2012; Du et al., 2013; Qin et al., 2014; Peters et al., 2016). Another study not included in this meta-analysis also found activation in the cerebellum (Matejko and Ansari, 2019). Our Control and RD + MD groups both activated vermis VI, left and right lobule VI during arithmetic processing compared to fixation (but not compared to active control). These were the same regions as those identified in the two groups during word processing in Study 1, and similarly, there were no differences in cerebellar activation during arithmetic processing between our RD + MD and Control groups (irrespective of which comparison task was used). Only one empirical study has examined functional activation during arithmetic in children with RD + MD (Peters et al., 2018). Most relevant to the current study, they found no differences between the group with RD + MD and controls on their arithmetic (subtraction) task, but several differences between the two groups during a non-symbolic subtraction task (dots), yet the cerebellum was not among them. In sum, the majority of studies of arithmetic in typical children do not show activation of the cerebellum and the only study of RD + MD to date found no differences for this group in the cerebellum. Therefore, our results of no between-group differences are consistent with the literature, even though we tackled the cerebellum more directly in our analysis.

Lastly, given the focus on the cerebellum in the context of low math skills, we next consider activation studies of MD. There are two meta-analyses for MD combining children and adults (Martinez-Lincoln et al., 2023; Tablante et al., 2023) that draw from 28 studies overall, and each meta-analysis reported altered right parietal lobe regions, but neither implicated the cerebellum. Of note, these meta-analyses included a range of task types (arithmetic, magnitude comparison, visual–spatial working memory, etc.) and one of them (Martinez-Lincoln et al., 2023) included several types of brain measures (activation, connectivity, and structure), but most were activation studies. When considering the original studies that were drawn on for these meta-analyses, eight used symbolic arithmetic tasks, specifically, (three included in Tablante et al., only, two included in Martinez-Lincoln et al., only, and three shared by both). Of these eight, only two studies observed differences in the cerebellum in MD, one reporting relatively less activation in the left cerebellum during complex and simple problems (Ashkenazi et al., 2012) and another reported relatively more activation in bilateral cerebellum during arithmetic verification (Iuculano et al., 2015). In sum, as for reading in RD, the majority of activation studies included in these meta-analyses do not report differences in the cerebellum in MD, consistent with our findings in the combined RD + MD group.

### 4.4 Functional connectivity of the cerebellum to cortical math-related regions

As in Study 1, in Study 2 we examined task-independent background functional connectivity and task-dependent functional connectivity, this time during an arithmetic processing task and focusing on connections between the cerebellum and regions known to be involved in arithmetic. As noted above, resting-state studies have demonstrated relationships the cerebellum has with fronto–parietal and ventral attention networks in adults (Buckner et al., 2011) and these overlap in their location not only with those associated with RD as mentioned above, but also with those associated with MD (Ashkenazi et al., 2013; Peters and De Smedt, 2018). Furthermore, a resting-state study in children has shown cerebellar FC with cortical regions such as angular gyrus and intraparietal lobule (Dosenbach et al., 2010). However, there are few studies in children that chose to make the cerebellum a region of interest, and none in children with RD + MD. Here we examined functional connections between the cerebellum and cortical regions known to subserve arithmetic to test for the anomalies due to math disability.

For background connectivity in Study 2, we found that all cerebellar seed regions had at least one positive connection with another region and most had one positive connection with a cortical target region in the Control group as well as in the RD + MD group. A positive functional connection for right crus I with left middle frontal gyrus was observed in both groups. Even though each group had other connections, there were no differences when comparing the Control and the RD + MD group for cerebellar-cortical intrinsic connections. However, the RD + MD group had relatively greater positive intrinsic functional connectivity within the cerebellum, between left and right lobule VIIb. Somewhat surprisingly, we did not find in this study (or in Study 1) background connectivity between cerebellar lobule VIIb/Crus II with intraparietal lobule, which was previously discovered with an analysis utilizing Neurosynth by Alvarez and Fiez (2018).

Prior studies have probed intrinsic FC between cortical regions in typically developing children. These seed-to-seed (Emerson and Cantlon, 2012) and seed-to-voxel (Jolles et al., 2016) analyses found intrinsic FC between parietal and frontal (as well as other) regions; and the strength of these connections was predictive of gains in numerical abilities (Evans et al., 2015). However, these studies did not include the cerebellum as a region of interest. As already noted above, one resting-state FC study has compared RD + MD children to RD-only, MD-only and Controls (Skeide et al., 2018), and found that RD + MD had reduced intrinsic connectivity between right para-hippocampal gyrus and right intraparietal sulcus in comparison to the other groups, but did not include the cerebellum in their region of interest analysis (Skeide et al., 2018).

When considering the literature in children with math disability, a seed-to-voxel study found more background functional connectivity between intraparietal sulcus seed and cortical regions (including bilateral superior frontal cortex), as well as the left cerebellum (including bilateral crus I and left crus II) in the group with MD relative to controls (Michels et al., 2018). Another seed-to-voxel study also found relative hyperconnectivity between left and right intraparietal sulcus seeds and the bilateral fronto-parietal network in children with MD (Jolles et al., 2016). Jolles et al. also used an alternative method (fractional amplitude of low-frequency) to explore intrinsic brain dynamics without *a priori* ROIs and found an increased aberrant fluctuation within bilateral cerebellum for the MD group when compared to the control group. Therefore, although Jolles et al., did not report cerebellar FC with the seed-to-voxel analysis, both studies found increased intrinsic connections of the cerebellum in the MD group relative to controls. In contrast, our results did not find significant differences between groups for cerebellar-cortical connectivity.

Turning to the gPPI FC analysis to test for task-dependent functional connections during arithmetic using the same cerebellar seed and cortical target regions, we found no task-dependent FC during arithmetic in the Control nor the RD + MD group and no between-group differences. Although no prior task-dependent connectivity studies have been performed in RD + MD children for arithmetic tasks, task-dependent connectivity studies on MD children are useful for the interpretation of this result. One such study reported hyper-connectivity between a seed in the intraparietal sulcus and multiple brain systems including the lateral fronto-parietal and default mode networks in children with MD during arithmetic (addition and subtraction) processing (Rosenberg-Lee et al., 2015). However, the cerebellum was not one of these regions included in this seed-to-voxel analyses.

### 4.5 Limitations and future studies

Taken together, this is the first study investigating cerebellar function in co-occurring reading and math disability. Our results are important in terms of understanding the brain-bases of these disorders as well as implications for treatment. We offer information on brain activity, task-independent background functional connectivity and task-dependent functional connectivity, all performed with special focus on the cerebellum. We found no differences between the group with RD + MD and controls on any of these measures. While our lack of a between-group difference is not unexpected given prior studies in RD or MD, it is important to discuss potential factors that may have led to the reported null results, including sample size, tasks, use of IQ as a covariate, and participants. One common concern is sample size. To ensure that the lack of results for activation of the cerebellum for reading or arithmetic when contrasted to the Active Control conditions was not due to insufficient statistical power, we conducted a *post hoc* analysis combining the two groups (Controls together with RD + MD group). For both real word Reading (*n* = 49) and Arithmetic (*n* = 30) relative to the Active Control tasks, we found no significant activation in the cerebellum. Moreover, we used Bayesian statistics to test for support of the alternative hypothesis and did not find it for any of the eight cerebellar sub-regions. Future studies in even larger samples would be beneficial to bolstering these findings. The tasks used here to elicit activation during reading and during arithmetic have been used previously by us and others. In our own work in children, we found them to induce robust activation in the cortex during reading (Turkeltaub et al., 2003; Olulade et al., 2013) and during arithmetic (Evans et al., 2014, 2016; Brignoni-Pérez et al., 2021); and both tasks have revealed between-group differences (Olulade et al., 2013; Evans et al., 2014). As such it is unlikely that the lack of task-specific cerebellar activation and between-group differences in the current study is related to these specific tasks. Our groups were not matched on IQ, and one may wonder if using IQ as a covariate in our analyses as we did, may have taken away from potential group differences, since IQ is correlated with our cognitive domains of interest. To address this possibility, we repeated our analyses without using IQ as a covariate, yet still found no between-group differences.

Our participants were users of an alphabetic writing system and the background literature presented here primarily reports on prior studies conducted with participants using alphabetic languages. However, as noted in the Introduction, there are reports of greater activity in the cerebellum (Feng et al., 2017; however not in Li et al., 2020); and stronger functional connectivity between the cerebellum and cortical regions known to subserve reading in Chinese children with reading disability relative to controls (Feng et al., 2017; Li et al., 2020). Future studies should investigate cerebellar involvement in children with RD + MD in logographic languages.

A challenge in the study of participants with learning disabilities is heterogeneity and even subtypes. It has been argued that the prevailing problem in RD entails poor phonological and orthographic processing, associated with left temporal–parietal and occipital-temporal regions, respectively, with poor phonological awareness being identified in the majority of children with dyslexia (Vellutino et al., 2004). While some have argued that RD can be accounted for by impairments in skill automatization due to abnormal cerebellar function, the prevalence of children with behaviors indicative of cerebellar dysfunctions (based on tests involving balance or fine manual skills) has been noted to be low among those with RD (Ramus, 2003; White et al., 2006). As such, even if there are cases of cerebellar anomaly in RD, they are likely to be in the minority and may not be detected in group analyses typically employed in brain imaging studies. Another challenge in studies of learning disabilities is that many, but not all participants will have received some intervention for their reading or math difficulties. While improvement or compensation mechanisms resulting from these efforts could obscure between-group differences, it has been noted that there is little evidence for robust, systematic brain-based changes following treatment in reading disability (Krafnick et al., 2022; Perdue et al., 2022). Even if our participants had received intervention, they were still significantly impaired in their skills, performing below the 16th percentile in reading and math.

While we set out to test for differences in the cerebellum associated with reading and differences associated with arithmetic, we did not have firm expectations as to whether these would be in the same or in separate regions of the cerebellum. The former seemed most likely given that theories on the role of the cerebellum in reading or math both focused on the same functional aspects of the cerebellum (e.g., automatization), and given the high comorbidity rate between RD and MD. While we would not have been able to attribute differences in our two groups for both tasks to the same neural populations if they had been found to be in the same region of the cerebellum, establishing whether and where such differences exist for both reading and arithmetic was an important first step, and we found this not to be the case. Also, neuroanatomical measures, such as gray matter volume, cortical thickness and white matter tracts (which were not included in our review of the literature) could also be added as measures in future studies of RD + MD.

## 5 Conclusion

We tested theories of cerebellar anomalies in children with combined reading and math disabilities (RD + MD, or dyslexia with dyscalculia). We compared functional activity and connectivity during word processing as well as during arithmetic processing. Using a region-of-interest analysis approach we examined the cerebellum, and also sub-regions of the cerebellum, and found no activity specific to reading or arithmetic processing in the RD + MD group or the Control group, and no between-group differences. There were also no between-group differences in task-independent (intrinsic) cerebellar-cortical functional connectivity for word processing or arithmetic. The same was true for functional connectivity specific to word processing or arithmetic processing. Overall, our results do not support the notion of cerebellar dysfunction in children with reading and math disabilities.

## Data availability statement

The raw data supporting the conclusions of this article will be made available by the authors, without undue reservation.

## Ethics statement

The studies involving humans were approved by Georgetown University Institutional Review Board. The studies were conducted in accordance with the local legislation and institutional requirements. Written informed consent for participation in this study was provided by the participants’ legal guardians/next of kin.

## Author contributions

GE and SA conceived and designed the study. GE and AM were involved in overall study logistics. GE, SA, and AM were involved in data collection. SA performed statistical analyses and together with GE drafted the manuscript. All authors contributed to the article and approved the submitted version.

## Footnotes

^1^ https://www.nitrc.org/
