# Supplementary material for: Activation and functional connectivity of cerebellum during reading and during arithmetic in children with combined reading and math disabilities
Source: Front Neurosci. 2024 Apr 29;18:1135166. doi: 10.3389/fnins.2024.1135166 (PMC11090247; doi:10.3389/fnins.2024.1135166)
Supplement: Supplementary file 1 [file Table_1.DOCX]

Supplementary Material

Activation and Functional Connectivity of Cerebellum during Reading and during Arithmetic in Children with combined Reading and Math Disabilities

Sikoya M. Ashburn, Anna A. Matejko, Guinevere F. Eden^*^

***Correspondence:**Guinevere Eden
[edeng@georgetown.edu](mailto:edeng@georgetown.edu)

# Supplementary Tables

Supplementary Table 1. Demographics and behavioral scores for Control and RD+MD groups in Study 1. All scores reported are averages of standard scores ± standard deviation. p-values listed for student t-Test for between-group differences, significance determined if p < .05. ‡ denotes tests from the Wechsler Abbreviated Scale of Intelligence (WASI); †denotes tests from the Woodcock Johnson-III (WJ-III).

|  | Control | RD+MD | *p*-value |
| --- | --- | --- | --- |
| N | 23 | 26 | *--* |
| Sex (F/M) | 13/10 | 12/14 | *--* |
| Age (years) | 9.7 ± 1.8 | 10.3 ± 1.4 | 0.232 |
| Verbal IQ‡ | 120.6 ± 14.5 | 102.4 ± 12.5 | <0.001 |
| Performance IQ‡ | 114.0 ± 13.1 | 101.6 ± 11.4 | 0.001 |
| Word ID† | 115.5 ± 12.4 | 75.8 ± 8.8 | <0.001 |
| Word Attack† | 110.4 ± 12.6 | 86.2 ± 9.6 | <0.001 |
| Math Fluency† | 106.9 ± 27.4 | 75.3 ± 8.3 | <0.001 |
| Calculation† | 116.3 ± 8.0 | 89.3 ± 12.4 | <0.001 |

Supplementary Table 2. Demographics and behavioral scores for Control and RD+MD groups in Study 2. All scores reported are averages of standard scores ± standard deviation. p-values listed for student t-Test for between-group differences, significance determined if p < .05. ‡ denotes tests from the Wechsler Abbreviated Scale of Intelligence (WASI); †denotes tests from the Woodcock Johnson-III (WJ-III).

|  | Control | RD+MD | | *p*-value | |  |
| --- | --- | --- | --- | --- | --- | --- |
| N | 16 | | 14 | | *--* | |
| Sex (F/M) | 6/10 | 6/8 | | *--* | |  |
| Age (years) | 10.1 ± 2.0 | 10.8 ± 1.3 | | 0.273 | |  |
| Verbal IQ‡ | 120.7 ± 14.3 | 97.5 ± 13.0 | | <0.001 | |  |
| Performance IQ‡ | 115.6 ± 11.1 | 103.8 ± 12.2 | | 0.012 | |  |
| Word ID† | 118.4 ± 10.5 | 79.4 ± 6.7 | | <0.001 | |  |
| Word Attack† | 113.2 ± 11.4 | 88.1 ± 5.8 | | <0.001 | |  |
| Math Fluency† | 105.5 ± 13.9 | 75.8 ± 9.6 | | <0.001 | |  |
| Calculation† | 114.7 ± 5.6 | 90.7 ± 13.3 | | <0.001 | |  |

**Supplementary Table 3. Participant in-scanner performance for Control and RD+MD groups during single word processing in Study 1.** Values reported as average ± standard deviation. p-values listed for student t-Test for between-group differences, significance determined if p < .05.

|  | Control | RD+MD | *p*-value |
| --- | --- | --- | --- |
| **Accuracy (% correct)** |  |  |  |
| Real Words | 90.1 ± 8.6 | 90.7 ± 8.5 | 0.797 |
| False Fonts | 90.2 ± 7.2 | 87.4 ± 11.1 | 0.298 |
| Real Word > False Font | -0.2 ± 5.7 | 3.3 ± 8.9 | 0.113 |
|  |  |  |  |
| **Response Time (ms)** |  |  |  |
| Real Words | 924.4 ± 139.8 | 1074.1 ± 192.2 | 0.004 |
| False Fonts | 927.3 ± 126.6 | 1089.8 ± 203.1 | 0.002 |
| Real Word > False Font | -2.8 ± 53.7 | -15.7 ± 103.5 | 0.589 |

Supplementary Table 4. Participant in-scanner performance for Control and RD+MD groups during arithmetic processing task in Study 2. Values reported as average ± standard deviation. p-values listed for student t-Test for between-group differences, significance determined if p < .05.

|  | Control | RD+MD | *p*-value |
| --- | --- | --- | --- |
| **Accuracy (% correct)** |  |  |  |
| Arithmetic | 84.2 ± 12.6 | 68.0 ± 14.3 | 0.003 |
| Active Control | 93.1 ± 6.5 | 82.3 ± 15.0 | 0.023 |
| Arithmetic > Active Control | -8.9 ± 11.3 | -14.3 ± 17.9 | 0.412 |
|  |  |  |  |
| **Response Time (ms)** |  |  |  |
| Arithmetic | 1850.5 ± 507.4 | 2045.0 ± 280.9 | 0.199 |
| Active Control | 1210.3 ± 233.2 | 1416.4 ± 401.4 | 0.107 |
| Arithmetic > Active Control | 640.2 ± 365.7 | 628.6 ± 531.0 | 0.987 |
